# Electroactive Biomaterials for Cardiovascular Tissue Engineering: Mechanisms, Design Strategies, and Therapeutic Applications

**DOI:** 10.3390/jfb17060295

**Published:** 2026-06-14

**Authors:** Jay Ming Tong, Dake Hao

**Affiliations:** 1 Department of Surgery, University of California Davis, Sacramento, CA 95817, USA; 2 Department of Biomedical Engineering, University of California Davis, Davis, CA 95616, USA; 3 Shriners Children’s—Northern California, Sacramento, CA 95817, USA

**Keywords:** bioelectrical cues, electroactive biomaterials, conductivity, piezoelectricity, cardiovascular tissue engineering

## Abstract

Cardiovascular diseases remain the leading cause of mortality worldwide, highlighting the urgent need for more effective therapeutic strategies. Despite substantial advances in conventional biomaterials, their limited ability to support functional integration and dynamically interact with the biological microenvironment continues to hinder therapeutic outcomes. Native cardiovascular tissues rely on tightly regulated bioelectrical signaling to coordinate cellular communication, tissue homeostasis, and functional repair. Consequently, recreating these bioelectrical cues has emerged as a key design principle in cardiovascular tissue engineering. Electroactive biomaterials have gained increasing attention as a promising platform to address this challenge by enabling electrical modulation of cellular behavior and tissue function. In this review, we summarize the intrinsic bioelectrical properties of cardiovascular tissues and discuss the roles of electrical stimulation in regulating disease-relevant cellular responses. We further highlight recent advances in the development of conductive, piezoelectric, and other electroactive biomaterials for cardiovascular tissue engineering applications. Finally, we critically discuss the major challenges and future opportunities in the field, including tissue-specific responses, stimulation parameter optimization, long-term safety, and clinical translation. Collectively, electroactive biomaterials represent a promising and rapidly evolving frontier for the development of dynamic, responsive, and next-generation therapies for cardiovascular diseases.

## 1. Introduction

Cardiovascular diseases (CVDs) have remained the leading cause of mortality worldwide for more than three decades, accounting for 20.5 million deaths in 2021, of which approximately 85% were attributable to myocardial infarction and stroke [1,2]. Driven by aging populations, sedentary lifestyles, and unhealthy dietary habits, global CVD-related mortality is projected to reach 23.6 million by 2030 [1,3,4], placing an enormous burden on both patients and healthcare systems [2,5]. Atherosclerosis, the primary underlying cause of CVDs, leads to progressive narrowing or occlusion of blood vessels, resulting in ischemia and myocardial infarction (MI) [6,7]. In cases of severe vascular obstruction, bypass surgery is often required to restore blood flow using vascular grafts [8,9,10]. Although autologous vessels remain the clinical gold standard, many patients lack suitable donor vessels due to pre-existing vascular disease or previous surgical procedures, necessitating the use of synthetic grafts. Following MI, coronary artery occlusion causes prolonged oxygen deprivation, cardiomyocyte necrosis, and subsequent fibrotic scar formation [11,12]. Compensatory neurohumoral activation initially acts to preserve cardiac output; however, sustained activation promotes maladaptive ventricular remodeling, ultimately leading to heart failure despite surgical and pharmacological interventions. Contemporary guideline-directed medical therapy for heart failure is based on four major therapeutic pillars, including angiotensin-converting enzyme inhibitors (ACEi), beta-blockers, mineralocorticoid receptor antagonists (MRAs), and sodium–glucose cotransporter 2 inhibitors (SGLT2i) [13,14]. In addition, device-based therapies are widely employed, including cardiac resynchronization therapy (CRT) to restore coordinated ventricular contraction, implantable cardioverter-defibrillators (ICDs) to prevent sudden cardiac death from malignant arrhythmias, and left ventricular assist devices (LVADs) to provide mechanical circulatory support. LVADs may serve as a bridge to recovery in patients with reversible myocardial dysfunction, a bridge to transplantation while awaiting a donor heart, or as destination therapy for patients who are not transplant candidates [15]. Despite these advances, heart transplantation remains the definitive treatment for end-stage heart failure, although its application is severely limited by donor organ shortages and the risk of immune rejection. To address these challenges, biomaterial-based cardiac patches capable of delivering cells, bioactive molecules, or therapeutic agents have emerged as promising strategies for mitigating adverse remodeling and promoting cardiac repair [16]. Despite significant advances, current approaches for cardiovascular tissue replacement and regeneration continue to face substantial challenges. Vascular grafts remain susceptible to thrombosis and intimal hyperplasia, leading to graft failure [6,16], while cardiac patches often suffer from limited biocompatibility, poor cell retention, insufficient integration with host tissue, and barriers to clinical translation [12,17]. These limitations have driven the development of cardiovascular tissue engineering (CVTE), which seeks to create advanced biomaterial platforms capable of more effectively restoring cardiovascular structure and function.

A key design consideration in CVTE is the recreation of the native electrical microenvironment, which plays a critical role in coordinating cellular excitation, contraction, and intercellular communication alongside biochemical and mechanical signaling cues [18]. Bioelectricity refers to endogenous electrical signals generated across the plasma membrane through the coordinated activity of ion channels, pumps, and transporters, regulating biological processes from the cellular to the organismal level [19,20]. These signals can subsequently be propagated between neighboring cells through gap junction-mediated electrical coupling [21]. Both cardiac and vascular tissues possess intrinsic bioelectrical properties that are essential for maintaining physiological function [22,23,24]. In the heart, electrical signaling governs cardiomyocyte alignment, excitation–contraction coupling, and synchronized tissue contraction [25]. Following MI, cardiomyocyte death and scar formation disrupt electrical signal propagation, resulting in impaired electromechanical function [11,12]. While early cardiac patches and injectable hydrogels were largely electrically inert, more recent designs have incorporated conductive biomaterials to restore electrical connectivity and improve functional integration with host myocardium [26]. Similarly, vascular tissues composed of endothelial cells (ECs), vascular smooth muscle cells (VSMCs), and fibroblasts are highly responsive to electrical cues. Numerous studies have demonstrated that electrical stimulation (ES) can enhance endothelial cell proliferation, angiogenesis, and vascularization [27,28,29,30], while also modulating the phenotype and function of VSMCs and fibroblasts [27,31]. Together, these findings underscore the importance of incorporating electrical functionality into biomaterial design for cardiovascular applications.

Electroactive biomaterials, including conductive and piezoelectric materials, offer unique opportunities to locally transmit or generate electrical signals to promote tissue repair and regeneration [32,33]. Unlike conventional pro-angiogenic drugs or growth factors, whose efficacy may be limited by diffusion and off-target effects, electroactive biomaterials can provide spatially localized and sustained electrical cues [32]. These materials may deliver electrical stimulation directly through external power sources or generate electrical signals autonomously through piezoelectric mechanisms that convert physiological movements into electrical outputs. The latter approach is particularly attractive due to its self-powered, minimally invasive nature and potential for long-term clinical implementation [32,34]. Conductive biomaterials have demonstrated considerable promise in restoring endogenous electrical signaling and enhancing cardiac tissue regeneration following injury [33]. Consequently, there has been growing interest in harnessing electroactive biomaterials and electrical stimulation strategies for cardiovascular repair and regeneration. In this review, we summarize the native bioelectrical properties of cardiovascular tissues, examine the therapeutic potential of electrical cues in modulating cellular responses during CVD progression, highlight recent advances in electroactive biomaterials for cardiovascular tissue engineering, and discuss the major challenges and future opportunities for their clinical translation.

## 2. Bioelectrical Properties and Signaling of Cardiovascular Tissues

### 2.1. Cardiac Tissue

**Figure 1:**
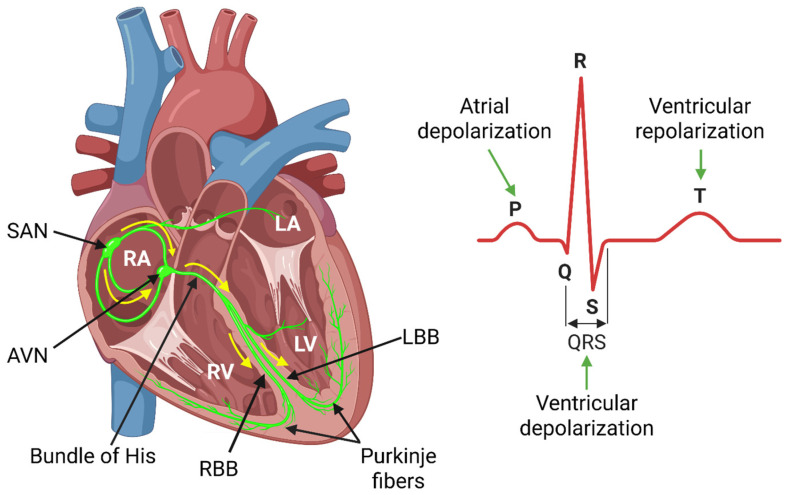
Electrophysiology of the heart. Schematic of heart conduction system with electrical signal starting from the sinoatrial node (SAN) to atrioventricular node (AVN), through the bundle of His, to left and right bundle branches (LBB and RBB), and lastly to the Purkinje fibers, triggering heart contraction (**left**). ECG of heart cycle during depolarization and repolarization (**right**). Image created with BioRender.com.

Heart function is governed by an electromechanical system composed of impulse-generating cells and a conduction pathway, as depicted in Figure 1. Synchronized electrical impulses originating from pacemaker cells in the sinoatrial node (SAN) travel to the atrioventricular node (AVN), down the bundle of His, and through the Purkinje fiber network in the ventricles, triggering ventricular cardiomyocyte contraction and blood ejection from the heart [22,35,36]. The cardiac cycle consists of diastole, the relaxation phase when the heart fills with blood, and systole, the contraction phase when the heart pumps blood throughout the body [37].

This electrical activity is reflected in the electrocardiogram (ECG), which consists of the (1) P wave during atrial depolarization and contraction, (2) QRS complex during ventricular depolarization and contraction, and (3) T wave during ventricular repolarization and relaxation. The heart also exhibits anisotropic conductivity of 1.6 × 10^−3^ S/cm longitudinally and 5 × 10^−5^ S/cm transversally [38].

CMs are the primary functional cells of the heart responsible for the electromechanical action. They undergo synchronized contraction and relaxation mediated by voltage-gated ion channels, including sodium (Na^+^), potassium (K^+^), and L-type calcium (Ca^2+^), to pump blood throughout the body, which is reflected by the 5 phases of the cardiac action potential [37,39]. Starting in phase 4 (resting potential), inward rectifier K^+^ channels (I_K1_) maintain the CM negative membrane potential at approximately −90 mV via K^+^ efflux [37,40]. In phase 0 (rapid depolarization), a stimulus from pacemaker cells or neighboring CMs raises the CM membrane potential past the −70 mV threshold, triggering opening of voltage-gated Na^+^ channels and rapid Na^+^ influx, raising the potential up to approximately +50 mV [41,42]. In phase 1 (early repolarization), Na^+^ channels close and transient outward K^+^ channels (I_to_) open to allow K^+^ efflux, lowering the membrane potential down to 0 to +30 mV [40,41]. In phase 2 (plateau), voltage-gated L-type Ca^2+^ channels open, allowing Ca^2+^ influx to balance K^+^ efflux, stabilizing the potential near 0 mV [39]. Ca^2+^ influx also triggers additional release of Ca^2+^ from the sarcoplasmic reticulum, initiating muscle contraction. In phase 3 (final repolarization), Ca^2+^ channels close while delayed rectifier K^+^ channels open, restoring resting membrane potential via K^+^ efflux [40,42]. Gap junctions located at interplicate regions of mature intercalated discs mediate intercellular electrical coupling and facilitate the propagation of actional potentials, enabling consistent electrical propagation and synchronized heart muscle contraction [43]. These electrical cues are vital for CM maintenance, alignment, gap junction formation, and cardiac marker expression in stem cells [25]. Thus, recapitulating the heart’s native bioelectrical environment is critical for developing advanced biomaterials to promote cardiac tissue regeneration.

### 2.2. Vascular Tissue

The vascular system also exhibits intrinsic bioelectrical properties, including electrical fields, piezoelectricity, transmembrane potentials, and ion channel activity, which are crucial for vascular function and cell regulation. Blood flow generates an electric field in blood vessels known as electrokinetic vascular streaming potential (EVSP) [44]. Sawyer et al. first measured streaming potentials in live rabbit aorta and vena cava in 1966 in the order of 5–10 mV using intravascular electrodes [45]. This phenomenon arises from the loose attraction between positive ions in the blood and the negatively charged endothelial glycocalyx layer (EGL), a brush-like vascular lumen lining composed of glycolipids, glycoproteins, and proteoglycans [46]. These interactions form the electric double layer (EDL) at the endothelial surface. As blood flow displaces positive counterions away from the EDL, the accumulation of positive ions downstream leads to ion imbalance and electric potential difference with the ions upstream, inducing an electric field in the vasculature known as EVSP [46]. Bergethon later demonstrated that EVSP-based extremely low-frequency (ELF) electric fields (0–2 Hz) modulate EC and VSMC function, enhance NO production, and alter pharmacological responses [44,47]. Moreover, vascular injury can generate a local positive charge in the negatively charged vascular intima, which has been associated with thrombosis [48].

Additionally, blood vessels exhibit piezoelectricity, the generation of electrical charge from mechanical stress, due to the presence of collagen and elastin in the extracellular matrix (ECM) of vessel walls [49,50]. Collagen, the predominant ECM protein, possesses anisotropic triple helix structure consisting mainly of glycine, proline, and hydroxyproline, which enables electric charge generation during vessel deformation or blood pressure changes [37,51,52]. Similarly, elastin, composed of tropoelastin and microfibrils, exhibits piezoelectric responses that may contribute to VSMC migration and proliferation [37,53]. Piezoelectricity was first reported in vessel walls by Fukada et al. in 1969 [54], a property of biological tissues stemming from shear-induced distortion of crosslinks in aligned, asymmetric fibrous molecules [55,56].

At the cellular level, vascular cells such as ECs, VSMCs, and fibroblasts express various ion channels, including calcium (Ca^2+^), sodium (Na^+^), potassium (K^+^), and chloride (Cl^−^) transporters, which regulate physiological processes, electrical signaling conduction, and cell membrane potential [23,57]. ECs are electrically coupled to neighboring ECs via homocellular gap junctions formed by connexins such as Cx37 and Cx40, and to overlying VSMCs via heterocellular gap junctions such as Cx43, which facilitate cell–cell communication to modulate vascular function [24,58]. Resting membrane potentials range from −51.6 ± 4.9 mV in ECs to −60 to −35 mV in VSMCs, which are mediated by Ca^2+^ and K^+^ channels [57,59,60]. ECs primarily express small- and intermediate-conductance Ca^2+^-activated K^+^ channels (SK_Ca_/IK_Ca_), whereas SMCs express voltage-gated K^+^ (K_V_) and large-conductance Ca^2+^-activated K^+^ (BK_Ca_) channels [57,61,62].

**Figure 2:**
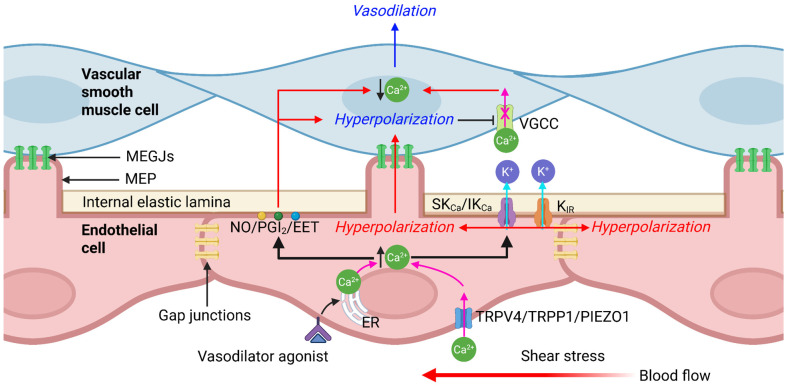
Endothelial electrical signaling generated by ion channels modulates vasodilation. Schematic of cell–cell communication via gap junctions between ECs and VSMCs in vasodilation. Shear stress and vasodilator agonists induce an increase in [Ca^2+^]_i_ and trigger hyperpolarization by K^+^ efflux. Hyperpolarization spreads along the endothelium and into VSMCs through myoendothelial gap junctions (MEGJs), leading to closure of VSMC voltage-gated Ca^2+^ channels (VGCCs) and decrease in VSMC [Ca^2+^]_i_. Elevated EC [Ca^2+^]_i_ also stimulates production of vasodilators such as NO, PGI_2_, and EETs. Together, they trigger VSMC relaxation and vasodilation through various pathways [58,63]. Image created with BioRender.com.

Electrical coupling between ECs and SMCs via ion channels influences vascular tone by promoting vasodilation, as shown in Figure 2, in a process known as endothelium-dependent hyperpolarization (EDH) [24]. The process starts with increased intracellular Ca^2+^ concentration ([Ca^2+^]_i_) in ECs via two primary pathways. First, shear stress from blood flow enables Ca^2+^ influx by activating mechanosensitive ion channels in ECs, including transient receptor vanilloid family member 4 (TRPV4), transient receptor potential polycystin family member 1 (TRPP1), and PIEZO1 channels [58]. Second, vasodilator agonists such as acetylcholine (Ach) trigger Ca^2+^ release from the endoplasmic reticulum (ER) by stimulating production of inositol 1,4,5 trisphosphate (IP_3_), which binds to receptors (IP_3_R) on the ER. Elevated [Ca^2+^]_i_ from internal and external sources activates the SK_Ca_/IK_Ca_ channels, causing K^+^ efflux and subsequent EC hyperpolarization, which also activates inner rectifier K^+^ (K_IR_) channels and further amplifies hyperpolarization. EC hyperpolarization propagating longitudinally along the endothelium over several mm via gap junctions is transmitted to adjacent VSMCs via myoendothelial gap junctions (MEGJs) located on myoendothelial projections (MEPs). VSMC hyperpolarization deactivates voltage-gated Ca^2+^ channels (VGCCs) and decreases Ca^2+^ influx. Since SMC contraction relies on Ca^2+^-calmodulin-mediated phosphorylation of myosin light chain (MLC), reduced [Ca^2+^]_i_ results in SMC relaxation, vasodilation, and increased blood flow. Elevated EC [Ca^2+^]_i_ also triggers production of EC-derived vasodilators such as nitric oxide (NO), prostacyclin (PGI_2_), and epoxyeicosatrienoic acids (EETs), which can induce VSMC relaxation through various mechanisms such as K^+^ efflux or Na^+^/K^+^-ATPase, contributing to hyperpolarization, reduced [Ca^2+^]_i_, and vasodilation [58,63]. In mouse studies, aging has been shown to impair electrical conduction along the endothelium due to current loss through SK_Ca_/IK_Ca_ channels, leading to reduced vasodilation and diminished blood flow [64]. The intrinsic electrical properties and responsiveness of vascular cells underscore the potential of leveraging electrical cues to guide vascular tissue regeneration in CVTE.

## 3. Electrical Modulation of Cellular Behavior During Cardiovascular Disease Progression

CVDs, such as myocardial infarction and atherosclerosis, involve a complex interplay between various cell types across different stages of progression. Electrical cues have emerged as a promising strategy to modulate cellular behavior and guide the design of biomaterials for CVD treatment.

### 3.1. Myocardial Infarction

**Figure 3:**
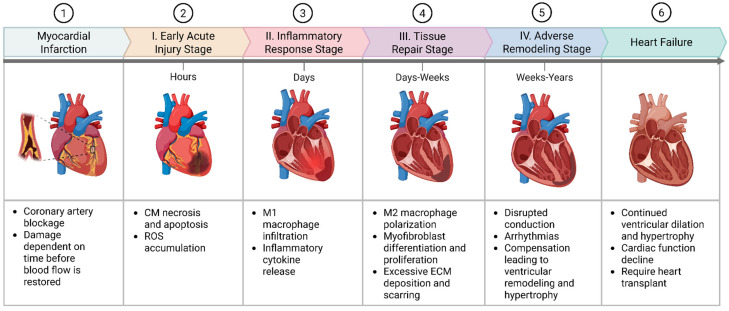
Schematic of myocardial infarction progression. MI transitions from (I) acute injury to (II) inflammatory response to (III) tissue repair to (IV) adverse remodeling, culminating in heart failure. Image created with BioRender.com.

MI, or a heart attack, results from the partial or complete blockage of blood flow in the coronary arteries, leading to oxygen deprivation and subsequent heart muscle damage and CM death. Even after restoring blood flow, the heart undergoes a series of pathological events, which can be categorized into 4 stages, as shown in Figure 3 [11,12]. First, in the early acute injury stage, ischemia leads to CM apoptosis and necrosis, reactive oxygen species (ROS) accumulation, and ventricular wall thinning. Next, during the inflammatory response stage, neutrophils and M1 pro-inflammatory macrophages phagocytose the dead cells and secrete inflammatory cytokines to promote angiogenesis. Subsequently, in the tissue proliferation and repair stage over several weeks, anti-inflammatory M2-like macrophages secrete high levels of pro-fibrotic cytokines including IL-10, IGF-1, FGF, PDGF, and TGF-ꞵ1 (which are also secreted by CMs, fibroblasts, platelets, vascular cells, and others in the infarcted heart [65]) inducing myofibroblast differentiation and excessive collagen deposition to replace the infarcted area with a non-conductive fibrotic scar, unlike native cardiac tissue with a conductivity of 1.5–1.7 × 10^−3^ S/cm [12,66]. Additionally, elevated matrix metalloproteinases (MMP-2, MMP-9) degrade the ECM, contributing to ventricular remodeling. Lastly, in the adverse remodeling stage, over weeks to years, the weak scar tissue results in sustained myofibroblast activation and collagen overproduction, reduced contractility, disrupted electrical conduction, and arrhythmias. Heart compensation through neurohumoral regulations leads to ventricular remodeling, hypertrophy, cardiac function decline, and eventually heart failure [12]. Electrical cues, delivered via external electrical stimulation (ES) or electroactive biomaterials, have been shown to restore native electrical conduction and promote cardiac regeneration, offering a targeted means to modulate pathological cellular responses throughout the MI progression [12,37].

#### 3.1.1. Inflammation Stage: Macrophages

The early stage of MI is characterized by inflammation driven by pro-inflammatory cytokines from M1 macrophages, which can exacerbate damage to surrounding healthy myocardium if left unresolved [12]. ES shows potential in modulating macrophage polarization between pro-inflammatory M1 and anti-inflammatory M2 phenotypes [67]. For example, Bianconi et al. applied 100 mV/mm direct current (DC) ES for 1 h daily over 3 days to THP-1-derived macrophages, which upregulated M2 markers (IL10, CD163, PPARG, TGM2, and CD206) in M0 and M1 macrophages and downregulated M1 marker CD86 in M1 macrophages [68]. In contrast, Gu et al. demonstrated square waveform ES promoted LPS/IFN-γ-induced M1 polarization of mouse bone marrow-derived macrophages due to activation of ion channels, which increased intracellular Ca^2+^ concentration and activated the NF-κB pathway [69]. Conversely, a sinusoidal waveform ES promoted both LPS/IFN-γ-induced M1 and IL4-induced M2 polarization due to an increase in intracellular Ca^2+^ as well as redistribution of TLR4 (M1) and IL-4Rα (M2) membrane receptors. Peng et al. also showed that electroacupuncture using 1 mA current intensity and 2/15 Hz frequency for 20 min promoted M2 macrophage polarization in MI rats after 7 days, downregulated inflammatory cytokines TNF-α, IL-1β, and IL-6, and exhibited cardioprotective effects [70].

#### 3.1.2. Regeneration Stage: Cardiomyocytes

Next, regenerating lost CMs is vital to restore normal cardiac function post-MI. ES has been widely used to promote cardiac stem cells differentiation for transplantation [71]. Ma et al. enhanced hiPSC cardiac differentiation via the Ca^2+^/PKC/ERK pathway using biphasic square wave pulses (5 ms, 5 Hz, 1 or 1.5 V/cm EF) for 1–30 days [72]. Pietronav et al. discovered biphasic square wave pulses (2 ms, 1 Hz, ±2.5 V, 3 days) upregulated early cardiac transcription factors (MEF2D, GATA4, Nkx2.5) and late cardiac sarcomeric proteins (troponin T, cardiac alpha actinin, SERCA2a) in cardiac progenitor cells more effectively than monophasic pulses [73]. Notably, the timing of ES is critical as early ES can disrupt CM differentiation, while late ES has diminished effects [71]. Zhang et al. also demonstrated that ES of cardiac mesenchymal stem cells (MSCs) with a square wave pulse (5 ms, 0.5 Hz, 1.5 V/1.8 cm) for 48–72 h enhanced extracellular vesicle (EV) secretion via nSMase2 activation, protecting CMs from hypoxia-induced apoptosis [74]. Furthermore, conductive biomaterials can support CM regeneration by enhancing endogenous electrical propagation even without ES [71]. Wang et al. used graphene sheets to accelerate hiPSC-CMs maturation, improving myofibril organization, conduction velocity, and Cx43 expression [75]. Similarly, Srinivasan et al. demonstrated that conductive bacterial nanocellulose-polypyrrole (PPy) patches increased cardiac marker expression in H9c2 rat cardiac myoblasts without chemical induction [76].

#### 3.1.3. Remodeling Stage: Fibroblasts

Lastly, regulating myofibroblast activation is essential to prevent scar formation, remodeling, and heart failure. In a rabbit MI model, He et al. reduced myocardial fibrosis and fibrotic markers (CTGF, α-SMA, TGF-ꞵ) using spinal cord stimulation (0.5 V, 0.2 ms pulse width, 50 Hz, 10 min on/30 min off, 2 weeks), potentially reducing the risk of arrhythmia and heart failure [77]. In a pig MI model, Mukherjee et al. applied localized high frequency stimulation (LHFS) (240 bpm, 0.8 V, 0.05 ms) from days 21 to 28 post-MI, which decreased MMPs, increased TIMPs (MMP inhibitors), and enhanced stiffness in the infarcted area, attenuating ventricular wall thinning and remodeling [78]. Similarly, AC stimulation (5 ms, 2 mA, 4 V/cm, 4 Hz, 24 h) of isolated pig fibroblasts reduced MMP-2 and MT1-MMP, while increasing TIMP-1, indicating direct modulation of fibroblast-mediated MMP activity. Somesh et al. also showed microcurrent treatment (1–2 μA/cm^2^) attenuated rat cardiac myofibroblast differentiation, reducing myofibroblast markers (α-SMA, SM22, and SMemb), collagen marker expression, TGF-β1 expression, and MMP-2 and MMP-9 expression, highlighting the potential of ES to reduce cardiac fibrosis for cardiac repair after MI [79]. These studies provide evidence of the therapeutic implications of ES in cardiac repair, demonstrating the ability to promote anti-inflammatory M2 macrophage polarization, enhance stem cell-derived CM maturation, and reduce myofibroblast activation. However, observed variations in outcomes across studies underscore the parameter- and context-dependent effects of ES. This suggests that optimal cardiac regeneration will require a tailored treatment strategy specific to the inflammatory, regenerative, and remodeling phases following MI rather than a single, uniform protocol applied throughout disease progression.

### 3.2. Atherosclerosis

**Figure 4:**
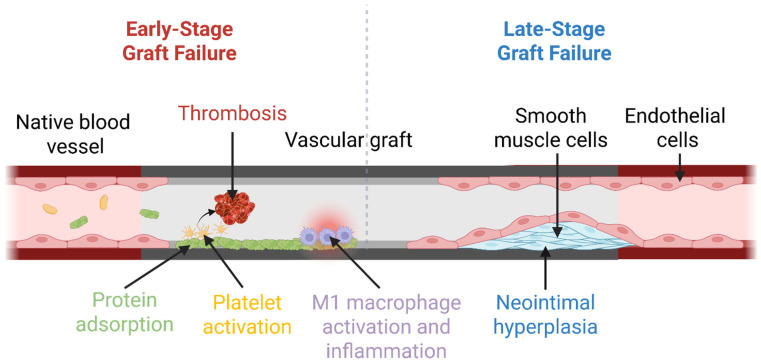
Schematic of vascular graft failure modes. Early-stage vascular graft failure can be caused by lumen occlusion due to thrombosis initiated by poor hemocompatibility, protein adsorption, and platelet activation. Additionally, persistent M1 macrophage-mediated inflammation during FBR impairs endothelialization and stimulates VSMC overproliferation, thereby increasing risk of thrombosis and neointimal hyperplasia and reducing long-term patency. Image created with BioRender.com.

CVDs, including coronary artery disease, peripheral arterial disease, deep vein thrombosis, cerebrovascular disease, ischemic heart disease, and stroke, are primarily caused by atherosclerosis, which is the chronic accumulation of lipids in blood vessels that restricts blood flow and leads to tissue damage from nutrient and oxygen deprivation [6,80]. Autologous and artificial vascular grafts are widely used in bypass surgeries to restore circulation. When autologous vessels are unavailable due to age, disease, or limited supply, artificial vessels are used. While medium- and large-diameter artificial grafts (>6 mm) achieve over 90% 5-year patency rate, small-diameter grafts (<6 mm) perform poorly with less than 50% 5-year patency rate [6,16]. Graft failure occurs through various modes, as depicted in Figure 4 [6,81]. Early failure is driven by the lack of an endothelial barrier, triggering protein adsorption, platelet adhesion and activation, and thrombosis [6,16,82]. Graft implantation also triggers the foreign body reaction (FBR) cascade, which is characterized by robust inflammation driven by M1 macrophages [83,84]. While short-term M1 macrophage activation promotes EC recruitment and tissue repair through secretion of pro-angiogenic cytokines, chronic inflammation prevents proper endothelialization and stimulates VSMC overproliferation, resulting in increased risk of thrombosis, intimal hyperplasia, calcification, and stenosis in the later stages. Other factors, including compliance mismatch and abnormal hemodynamics, further exacerbate SMC proliferation and ECM deposition, reducing vessel patency [6]. Emerging evidence indicates that electrical cues can modulate vascular and blood-related cells, such as platelets, macrophages, ECs, SMCs, and fibroblasts, thereby reducing graft failure and facilitating vascular tissue regeneration.

#### 3.2.1. Early Thrombosis and Inflammation Stage: Platelets and Macrophages

Following graft implantation, poor hemocompatibility can trigger platelet activation and thrombosis. Additionally, inflammation caused by M1 macrophage activation during FBR can lead to downstream complications [18,85,86]. Applied electric fields (EFs) can regulate platelet deposition at the site of vascular injury [87]. Cathode EFs (−13.0 mV/mm) reduce platelet deposition, while anode EFs (+16.1 mV/mm) enhance it, due to the electrostatic interactions with the negatively charged sialic acid on platelets. Studies from the 1950s to 1970s demonstrated that electrical signals can either promote or inhibit thrombosis, where an electrical potential over +300 mV resulted in blood cell aggregation [87,88]. The experimental setup consisted of electrodes immersed in platelet-rich plasma diluted in a buffer solution with an anticoagulant. Platelet precipitation onto the electrodes at different electrical potentials was observed through a microscope. Additionally, ES can induce macrophage polarization from a pro-inflammatory M1 to an anti-inflammatory M2 phenotype as previously discussed, modulating inflammation to enhance vascular regeneration and graft integration.

#### 3.2.2. Endothelialization Stage: Endothelial Cells

Next, endothelialization of the vascular graft is essential for maintaining a non-thrombogenic layer for long-term graft patency. ES can promote EC proliferation, migration, and elongation via activation of the vascular endothelial growth factor (VEGF) pathway [27,62,89]. Direct current (DC) ES (75–100 mV/mm) directly activates VEGF receptors and downstream PI3K/AKT and Rho/ROCK pathways, enhancing EC reorientation, elongation, and motility [28,90,91]. Using an electronic blood vessel with 50 mV/mm DC stimulation, Cheng et al. significantly enhanced human umbilical vein endothelial cell (HUVEC) migration and proliferation over 2 days in vitro, accelerating endothelialization of the vascular graft [92]. Additionally, ES can promote angiogenesis by stimulating fibroblasts to secrete fibroblast growth factor 2 (FGF2) via the nitric oxide synthase (NOS) pathway, activating MAPK/ERK signaling and increasing VEGF expression in ECs [29].

#### 3.2.3. Late-Stage Neointimal Hyperplasia: Vascular Smooth Muscle Cells

In late-stage graft failure, VSMC overproliferation drives neointimal hyperplasia and graft occlusion. ES can modulate restenosis by shifting VSMCs from a synthetic (vascular remodeling and uncontrollable proliferation) to a contractile phenotype (vascular maintenance) [93]. Rowlands et al. achieved VSMC phenotype switch by applying sinusoidal AC stimulation (50 μA, ±0.6 V, 5 and 500 Hz, 24–96 h) on VSMCs cultured on conductive PPy films coated with collagen IV and Matrigel [94]. Similarly, Derhambakhsh et al. showed 1000 μA sinusoidal stimulation (10 Hz, 20 min/day, 4 days) downregulated synthetic markers (ꞵ-actin, nestin, vimentin) and upregulated contractile markers (SMMHC and α-SMA) in VSMCs, likely via activation of the RhoA/ROK/myocardin pathway from Ca^2+^ influx through voltage-gated Ca^2+^ channels [93]. SMMHC and α-SMA (a marker also expressed in activated myofibroblasts) were selected due to lower expression levels in synthetic compared to contractile VSMCs. In another study, Zhang et al. demonstrated DC electric fields (3–4 V/cm, 30 min/day, 1–4 weeks) inhibited neointimal hyperplasia in a rabbit abdominal aorta balloon injury model through upregulation of the PTEN/p27Kip1 pathway, reducing SMC proliferation and type I collagen expression [95]. Furthermore, compliance mismatch between the native vessel and the implant has been associated with intimal hyperplasia due to flow disruption [96], as well as disrupting the stable performance of bioelectronic interfaces [97]. The integration of electroactive biomaterials in soft bioelectronics can address the compliance mismatch with cardiovascular tissues. For example, encapsulation of a piezoelectric sensor in an ultrathin, flexible polymer composed of polyimide and PDMS enabled vascular graft monitoring without affecting the implant’s mechanical properties [98]. Additionally, conductive hydrogels and intrinsically stretchable conductors can be used to reduce the mechanical mismatch between soft and hard interfaces, while bioadhesive layers can enhance interfacial adhesion to prevent delamination during cyclic mechanical loading [97].

## 4. Electroactive Biomaterials in Cardiovascular Tissue Engineering

**Figure 5:**
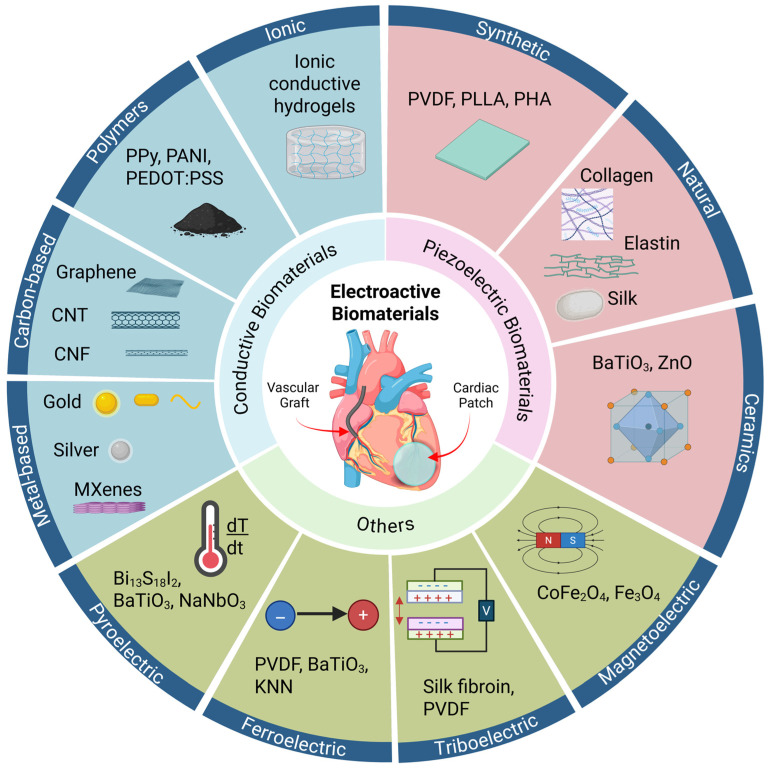
Schematic of electroactive biomaterials. Various types of conductive, piezoelectric, and other electroactive biomaterials used in cardiovascular tissue engineering and biomedical applications. Image created with BioRender.com.

Building on the intrinsic electrical responsiveness of cardiovascular tissues and cells, electroactive biomaterials hold great promise for replicating the native bioelectrical microenvironment, delivering targeted electrical cues, and modulating cellular behavior to promote tissue regeneration. The following sections discuss various types of electroactive biomaterials utilized in CVTE, as illustrated in Figure 5.

### 4.1. Conductive Biomaterials in CVTE

MI leaves a non-conductive scar over the infarcted area that disrupts native cardiac electrical signaling, contributing to arrhythmias and heart failure. Restoring the electrical connectivity is crucial for promoting cardiac regeneration, preventing ventricular remodeling, and restoring cardiac function [26]. Furthermore, conductive biomaterials can also promote endothelial cell proliferation and support vascularization, highlighting their potential in vascular tissue engineering [99,100]. Conductive biomaterials, even without ES, can regulate cell adhesion, proliferation, self-renewal, and differentiation, which is useful for guiding tissue regeneration [38]. The following sections summarize several types of conductive biomaterials used in CVTE, including metal-based, carbon-based, conductive polymers, and ionic conductive biomaterials.

#### 4.1.1. Metal-Based Conductive Biomaterials

Metals exhibit high mechanical strength, excellent electrical conductivity (10^5^–10^7^ S/m) due to the presence of free valence electrons, and ease of modification with bioactive agents [36]. Metal-based nanomaterials have been integrated into tissue scaffolds to improve electrical conductivity and mechanical properties [36,38].

Gold nanomaterials (AuNMs) have been extensively used in cardiac scaffolds for their high conductivity, erosion and oxidation resistance, biocompatibility, ease of fabrication, and tunable properties [26,36]. While bulk gold exhibits a conductivity of 4.11 × 10^7^ S/m, AuNMs show size-dependent conductivity, with 2 nm and 4 nm AuNPs thin films exhibiting intrinsic conductivities of 8.3 S/cm and 86 S/cm, respectively [101,102]. Mozaffari et al. added AuNPs into electrospun polyurethane/collagen scaffolds for potential use as a cardiac patch, achieving high hydrophilicity (59 ± 0.6°), mechanical strength (Young’s modulus: 1.53 ± 0.07 MPa), and enhanced HUVEC viability [103]. Sesena-Rubfiaro et al. showed AuNRs embedded into fibrin hydrogels supported long-term iPSC-CM maturation and viability for over 9 months in PDMS milli-tug devices compared to 1 month in control [104]. Tian et al. also demonstrated collagen/AuNW cardiac patches improved H9c2 rat cardiac myoblast proliferation without signs of cytotoxicity [105]. In vivo studies reported reduced infarct sizes in animal MI models. Qiu et al. incorporated biosynthesized AuNPs from *Staphylococcus aureus* into a Dopa-based GelMA/PEGDA cryogel with a conductivity of 3.282 × 10^−4^ ± 8 × 10^−5^ S/m, which enhanced CM attachment and contraction, and improved cardiac function in a rat MI model by decreasing infarct area [106]. Dong et al. coated decellularized heart ECM/silk fibroin (SF) scaffolds with AuNPs using EDC-NHS click chemistry, which had a conductivity of ~0.15 S/m (based on plot) and decreased infarct size from 89% to 65% in the rat MI model after 28 days [107]. While Au is largely biocompatible and nonimmunogenic, Au exhibits size- and shape-dependent toxicity and long-term safety concerns [36]. Smaller-sized NPs exhibit higher toxicity due to interference with biomolecules, higher surface to volume ratio, and induction of ROS generation, leading to pathological response [108]. For example, while 10 nm and 20 nm AuNPs induced heart muscle congestion, dilated vessels, RBC extravasation, and inflammation in rat hearts, 50 nm AuNPs resulted in only a small number of lymphocytes [108]. Various studies have also evaluated shape-dependent toxicity of AuNPs, with one study showing the highest toxicity in nanorods followed by nanostars and nanospheres [109], while another study showed the highest toxicity in nanospheres and nanorods compared to nanostars, nanoflowers, and nanoprisms [110], although size, concentration, exposure duration, surface properties, and cell type are also important considerations.

Silver is another commonly used metal, featuring higher conductivity than gold (6.30 × 10^7^ S/m) [101]. Silver nanoparticles (AgNPs) possess antimicrobial and anti-inflammatory properties and have been added to various scaffolds to enhance conductivity [36]. Allison et al. showed electrospun collagen/AgNP scaffolds with a conductivity of 8 × 10^−7^ S/m enhanced neonatal rat ventricular myocyte proliferation and Cx43 expression under ES (1 V, 5 ms, 5 Hz, 24 h), inhibited *Pseudomonas aeruginosa* biofilm formation, and avoided macrophage activation [111]. Similarly, Wickham et al. developed a collagen/AgNW scaffold that supported embryonic chicken cardiomyocytes proliferation and inhibited growth of *Escherichia coli* and *Staphylococcus epidermidis* bacteria [112]. Notably, lower AgNW concentration (0.1 mg/mL) promoted embryonic CM proliferation until day 7, whereas higher AgNW concentrations (0.5–1 mg/mL) decreased proliferation after day 5. Despite their benefits, AgNPs exhibit similar size-, concentration-, exposure-, and cell-dependent toxicity as AuNPs [113]. AgNPs can disrupt cardiac electrophysiology by inhibiting inward rectifying Na^+^ (I_Na_) and K^+^ (I_K1_) current channels, causing loss of CM transmembrane potential and excitability as well as lethal bradyarrhythmia in vivo [114]. The toxicity was attributed to direct effects of AgNPs rather than Ag^+^, ROS, or membrane injury.

MXenes are a novel class of 2D conductive nanomaterials composed of transition metal carbides, nitrides, or carbonitrides, with the general formula M_n+1_X_n_T_x_ [115]. MXene films exhibit high conductivity (2–2.4 × 10^4^ S/cm), hydrophilicity, and biocompatibility, making them promising candidates for bioelectronics and electroactive tissue repair [116,117]. Basara et al. cultured hiPSC-CMs on 3D printed titanium carbide (Ti_3_C_2_T_x_) MXene-PEG hydrogels, which upregulated cardiac markers (MYH7, SERCA2, TNNT2) and improved synchronous beating [118]. Wang et al. developed a highly biocompatible MXene (Ti_3_C_2_T_x_)/polyvinyl alcohol (PVA)/polyacrylamide (PAM) composite hydrogel with excellent mechanical strength (tensile strength: 140 kPa, 1296% elongation, 15 kPa adhesion strength) and conductivity (4.11 S/m) [119]. Yu et al. incorporated MXene (Ti_3_C_2_) into a methacrylated silk fibroin/hyaluronic acid hydrogel (0.32 S/m conductivity), which improved myocardial repair and cardiac function by increasing left ventricular ejection fraction (LVEF) and fractional shortening (LVFS) [120]. Ren et al. further demonstrated MXene/ECM hydrogels can eliminate ROS after MI, which decreased myocardial cell apoptosis and restored cardiac function [121]. Despite promising results, long-term safety, stability, and degradation of MXenes remain unclear [117]. Cytotoxicity varies with surface modification, synthesis method, size, dose, and exposure time [122]. Additionally, MXenes are prone to degradation and oxidation in physiological and humid environments, leading to loss of electrical conductivity [117]. This instability is particularly problematic in the highly dynamic and oxidative microenvironment of the infarcted myocardium, as well as in industrial scaling and storage. Further optimization is needed before MXenes can be translated into clinical applications.

#### 4.1.2. Carbon-Based Conductive Biomaterials

Carbon-based biomaterials, including 3D graphite, 2D graphene/carbon nanosheets, 1D carbon nanotubes (CNTs)/nanofibers (CNFs)/nanohorns, and 0D carbon dots/fullerene/nanodiamonds, have been widely used in cardiac, muscle, and nerve tissue engineering to increase mechanical strength and conductivity [26,38]. Carbon-based materials exhibit high conductivity (10^7^–10^8^ S/m), large surface area for bioactive compound loading, and tunable properties, depending on their form [36,38]. Among them, graphene derivatives such as graphene oxide (GO) and reduced graphene oxide (rGO), as well as CNTs, are most commonly used in cardiac tissue engineering due to their chemical stability, high conductivity, and large surface area [38,123].

Graphene exhibits high conductivity, but low hydrophilicity, limiting its applications [123]. Oxidizing graphene to produce GO improves hydrophilicity but reduces conductivity. To balance both properties, rGO was developed through chemical or thermal treatment of GO. Arıcı et al. developed 3D printed carboxymethyl cellulose (CMC)/gelatin (Gel)/GO cardiac patches crosslinked with EDC/NHS, achieving high stability, flexibility (61.03% strain), appropriate conductivity (7.0 × 10^−3^ S/cm), and enhanced H9c2 rat cardiac myoblast proliferation [124]. To replicate anisotropic conduction of the myocardium, Zhao et al. electrospun rGO/silk fibroin (SF) fibers into aligned (A) and random (R) orientations, which exhibited higher conductivity in the parallel than perpendicular direction, ranging from 0.2 to 0.3 S/cm [125]. The rGO/silk_A/R_ cardiac patches improved therapeutic outcomes in MI rats based on enhanced cardiac pumping function, LV wall thickening, CM survival, and angiogenesis, as well as reduced infarct size and arrhythmia susceptibility compared to random or nonconductive controls. However, the GO oxidation process can lead to batch variability and poor reproducibility. To address this, Wang et al. functionalized graphene with methoxytriethylene glycol (TEG-GR), enhancing solubility, hydrophilicity, immune camouflage, and electron migration [126]. TEG-GR was incorporated into dopamine-modified gelatin (GelDA) to fabricate self-adhesive cardiac patches, achieving conductivity up to 0.624 S/m. In rats, these patches improved ejection fraction (50.7%), reduced QRS interval (17.3%), and increased vessel density (78.0%) by restoring gap junctions, promoting angiogenesis, and suppressing CM apoptosis.

CNTs, formed by rolling graphene sheets into cylindrical structures, exist as single-walled (0.4–2 nm diameter) or multi-walled (MW) (2–100 nm diameter) structures [36]. MWCNTs can promote contraction and electrical activity in neonatal rat CMs by upregulating sodium/calcium exchanger 1 (NCX1) and gap junction formation [127]. Wang et al. developed an injectable, conductive shape-memory cardiac patch using methacrylated elastin, gelatin, and MWCNTs (20 mg/mL), exhibiting high conductivity and flexibility, enabling minimally invasive delivery with fibrin glue [128]. Implantation into rat and pig MI models improved LVFS and LVEF. To minimize cytotoxicity, Shokraei et al. electrosprayed low concentrations of MWCNTs (2–6 wt%) onto electrospun polyurethane nanofibers, achieving conductivity from 5.46 × 10^−5^ to 2.13 × 10^−2^ S/cm) while maintaining high cytocompatibility with H9c2 rat cardiac myoblasts and HUVECs [129]. Sun et al. formed hydrogels by combining temposulfonated CNTs (SCNTs) with TEMPO-oxidized cellulose nanofibrils (TOCNs), fielding conductivity ranging from 5.2 × 10^−6^ to 6.2 × 10^−2^ S/cm at 0–5 wt% SCNTs [130]. TOCNs provide excellent mechanical and biological properties and water dispersibility, while sulfonation prevents CNT aggregation and preserves conductivity. At just 1 wt% SCNT, the hydrogel promoted H9c2 rat cardiac myoblast proliferation and upregulated Cx43 expression.

CNFs, formed by stacked graphene layers, feature lower cytotoxicity and cost than CNTs [36,131]. Mehrabi et al. developed an electrospun CNF/gelatin cardiac patch with a conductivity of 8.39 ± 1.2 × 10^−5^ S/m, which upregulated CM markers (TrpT-2/TNNT2, ACTN4, Cx43) and promoted angiogenesis after subcutaneous implantation in mice [132]. Despite promising properties, graphene-based materials are non-biodegradable and exhibit potential toxicity depending on dose, size, shape, and fabrication process, which can accumulate over time [133]. While strategies such as polymer blending, PEGylation, functionalization, and size adjustment can improve biocompatibility, further evaluation is required before clinical use.

#### 4.1.3. Conductive Polymers

Conductive polymers possess conjugated double bonds throughout their polymer matrix, which enables delocalization of free valence electrons and conductivity ranging from 10^−7^ to 10^3^ S/cm [36,38]. Their conductivity can be tuned based on size, functional groups, and dopants via surface functionalization techniques. Some common conductive polymers used in biomedical applications include polypyrrole (PPy), polyaniline (PANI), and poly(3,4-ethylene dioxythiophene) polystyrene sulfonate (PEDOT:PSS) [26,38]. However, due to their brittleness and poor processability, conductive polymers are often blended with other biocompatible polymers for tissue engineering [36].

PPy is one of the most extensively studied conductive polymers used in tissue engineering, biosensors, and drug delivery, exhibiting high conductivity (10^2^–7.5 × 10^3^ S/cm), good biocompatibility, chemical stability, and stimuli-responsiveness [134]. However, its non-thermoplastic nature and brittleness limit its processability, requiring blending with other materials for tissue engineering applications [135]. Yin et al. fabricated silk fibroin/PPy (SP50) patches with conductivity up to 11.22 ± 0.63 × 10^−4^ S/cm, which enhanced CM protein markers (α-actinin, Cx43, cTnT) under in vitro ES (rectangular, 2 ms, 5 V/cm, 1 Hz) [136]. Implantation of the SP50 patch with matured CMs into MI rats improved LVEF, LV remodeling, and electrical signal propagation. Conversely, Srinivasan et al. demonstrated conductive bacterial nanocellulose/PPy patches (10^−5^–10^−2^ S/cm) partially differentiated H9c2 rat cardiac myoblasts into CMs without chemical induction or ES, and showed a synergistic effect with differentiation medium [76]. Recently, Li et al. designed a conductive, bioadhesive hydrogel for cardiac repair by incorporating polydopamine-PPy (PDA-PPy) NPs with aldehyde-modified Pluronic F127 (F127-CHO), dopamine-functionalized gelatin (GelDA), and astragaloside IV (AST) [137]. PDA-PPy promoted CM maturation via electrical coupling, while AST release improved cardiac function in MI rats by inhibiting CM ferroptosis through Nrf2/HO-1 upregulation. Furthermore, PPy scaffolds can modulate vascularization, hemocompatibility, and inflammation. W. Xiong et al. developed a hybrid PPy/holey graphene oxide-incorporated poly-(hydroxyethyl methacrylate) (p(HEMA)) cryogel with conductivity of 0.071 ± 0.013 S/m, which supported vascularization from seeded ECs, rebuilt vascular anastomoses in MI rats, and facilitated paracrine signaling for MI repair [100]. G. Xiong et al. coated PCL fibers with heparin-doped PPy, achieving low RBC hemolysis (3.9 ± 2.1%), improved anticoagulation, and prevented platelet activation or leukocyte attachment under 100 Hz AC stimulation [138].

PANI is another widely used conductive polymer, with good biocompatibility, electroactivity, commercial availability, affordability, and ability to be switched between conductive and resistive states [36,134]. The conductivity of PANI ranges from 30 to 200 S/cm, with emeraldine being the most conductive and stable form [134]. Similar to PPy, PANI is limited by poor processability, low flexibility, non-biodegradability, and risk of chronic inflammation, necessitating chemical modifications or composites to improve biocompatibility [36]. PANI composites with biodegradable polymers can enhance cardiac cell proliferation, alignment, and differentiation [135]. Bertuoli et al. showed that coaxially electrospun core-shell nanofibers with a PLA/PANI core and a PLA or PLA/PEG shell improved biocompatibility by preventing PANI release, while modulating cardiac cell adhesion, orientation, and morphology, and maintaining CM for 7 days [139]. Leite et al. developed a collagen/hyaluronic acid/silk fibroin/PANI cardiac patch that improved LV remodeling in a rat MI model, reducing infarct size, increasing LV thickness, and increasing LVEF and FS, despite minor hepatotoxicity and pro-oxidant effects [140]. Yu et al. engineered a PANI-based chronological adhesive hydrogel patch (CAHP) integrating mechanophysiological monitoring and electrocoupling therapy to treat MI [141]. Composed of functionalized-PANI (borate and carboxyl side chains) and polyvinyl alcohol (PVA), the CAHP adhered strongly to the epicardium within 10 min, avoided nonspecific adhesion, and exhibited a conductivity of 1.05–1.38 S/m. In MI rats, the CAHPs restored electrical propagation, maintained LV wall thickness, increased blood vessel density from 6.1% to 10.2%, and enabled cardiac monitoring through current change. PANI scaffolds can also promote EC adhesion and proliferation for vascular tissue engineering [142,143]. Li et al. demonstrated that electrospun PCL/PANI fibers, exhibiting a conductivity of 6.71 × 10^−3^ S/cm, better promoted HUVEC proliferation compared to PCL fibers, especially under ES (200–400 mV/cm) [142]. The same group also showed electrospun polyurethane (PU)/PANI fibers with a conductivity of 4.66 × 10^−2^ S/cm enhanced HUVEC proliferation even without ES, and demonstrated anticoagulant effects and high hemocompatibility as evidenced by low hemolysis (0.14%), long plasma recalcification time (123 s), and low platelet adhesion (6.87 × 10^5^ vs. 15.63 × 10^5^ cells/cm^2^ for PU) [143]. Overall, PANI is a well-documented conductive biomaterial for CVTE.

PEDOT, and its water-soluble form PEDOT:PSS, is a polythiophene (PTh) derivative that has recently gained attention in cardiac tissue engineering due to its biocompatibility, conductivity, and versatility [36,134]. While PEDOT:PSS has low conductivity (1 S/cm) due to its insulating PSS shell, various processing techniques can enhance its conductivity up to 4700 S/cm or higher [144,145]. However, limited stability in physiological environments due to PSS swelling and uncertain safety of additives used to increase conductivity remain challenges requiring further optimization [146,147]. Nevertheless, PEDOT:PSS continues to hold promise in tissue engineering and biosensor development, and has been incorporated into various hydrogel systems such as collagen, alginate [148], polyvinyl alcohol (PVA) [149], and gelatin [150,151] for cardiac tissue engineering. Roshanbinfar et al. developed a biohybrid collagen/alginate hydrogel with PEDOT:PSS with a combined ionic and electron-based conductivity (up to 35 ± 11 × 10^−4^ S/cm), which supported synchronous CM beating (220.6 bpm by day 11), maturation (alignment, sarcomere organization, Cx43 expression), and hiPSC-CM differentiation (1.9 µm near adult sarcomere length, enhanced beating frequency) [148]. Sauvage et al. created a conductive (~40 S/cm), stretchable (50%) bioactive PVA/PEDOT:PSS hydrogel functionalized with N-cadherin mimic peptide to promote cardiac fibroblast adhesion and proliferation, while inhibiting *Staphylococcus aureus* biofilm formation [149]. Furlani et al. constructed a gelatin/PEDOT:PSS hydrogel stabilized via dehydrothermal treatment without the use of crosslinking agents, exhibiting conductivity over 100 nS/cm, cardiac tissue stiffness (40–60 kPa), and enhanced H9c2 rat cardiac myoblast proliferation [150]. Recently, Chou et al. presented an injectable GelMA/PEDOT:PSS hydrogel that restored cardiac function and inhibited ventricular arrhythmias in a rabbit heart failure model [151]. A bioelectronic cardiac patch was then developed by integrating the hydrogel with an organic electrochemical transistor (OECT), enabling controlled release of loaded EVs over 24 h under applied voltage as well as biosensing capabilities for potential treatment and monitoring of MI. Moreover, PEDOT:PSS can influence the proliferation, attachment, and migration of non-excitable cells such as ECs by modulating membrane potential and ion transport. Mahmoudinezhad et al. showed 1% PEDOT:PSS in 5% gelatin/30% alginate hydrogels significantly promoted HUVEC proliferation after 7 days, enhanced nitric oxide release, and increased expression of EC markers (PECAM-1/CD31, KDR, VE cadherin, vWf), supporting its application in endothelial regeneration [99].

#### 4.1.4. Other Conductive Biomaterials

Silicon is a conductive metalloid with both metal and nonmetal properties. Silicon-derived nanomaterials, with their mesoporous structure and tunable electroconductivity through doping, have been utilized for various drug delivery and tissue engineering applications [26]. Richards et al. demonstrated silicon NWs combined with hiPSC cardiac spheroids and ES synergistically enhanced cell junction formation, improved contractility, and reduced spontaneous beating, showing potential for cardiac cell therapy [152]. Similar to other conductive materials, the cytotoxicity, biodegradation, and clearance of silicon require further investigation [26].

Ionic conductive biomaterials, which transfer ions rather than electrons, are another class of conductive materials used in myocardial repair [123]. Unlike traditional electroconductive materials, which suffer from aggregation, discontinuous conductivity, and toxicity, ionic conductive hydrogels provide homogenous, continuous conductivity even under strain [26]. Ionic conductive hydrogels can be classified into 3 types: electrolyte, polyelectrolyte, and ionic liquid conductive hydrogels [153]. Electrolyte conductive hydrogels utilize inorganic salt ions such as NaCl, KCl, LiCl, and AlCl_3_. Notably, AlCl_3_ exhibits cytotoxic effects, including cardiotoxicity, neurotoxicity, nephron toxicity, and hepatotoxicity due to ROS generation leading to oxidative stress, mitochondrial induction, and lipid peroxidation [154]. Polyelectrolyte hydrogels contain ionizable macromolecules and can be natural, such as alginate, chitosan, and hyaluronic acid, or synthetic, such as poly(2-acrylamido-2-methy-1-propanesulfonic acid) (PAMPS), poly(diallyldimethylammonium chloride) (PDAC), poly(styrene sulfonate) (PSS), and polyacrylic acid (PAA). Ionic liquids are a class of molten salts composed of cations, anions, or both bonded to a polymer backbone that remain liquid at room temperature [155]. Song et al. developed a self-healing ionic conductive hydrogel (POG_1_) for myocardial repair using polyacrylic acid (PAA), oxidized alginate (OA), and gelatin, exhibiting comparable ionic conductivity to native heart tissue (35.36 ± 7.72 × 10^−3^ S/cm) [156]. POG_1_ hydrogels outperformed PPy, CNT, and rGO hydrogels in promoting CM sarcomeres orientation and improved cardiac function in the rat MI model. More recently, Li et al. designed a mechanically robust hydrogel (CS/TA@PAs-Eu) for myocardial repair using chitosan (CS), lipoic acid (TA), proanthocyanidins (PAs), and Eu^3+^, which possessed an ionic conductivity of 1.3 × 10^−4^ S/cm [157]. The TA, PAs, and Eu^3+^ synergistically improved mitochondrial function and conductivity, antioxidation, and vascularization, respectively, and inhibited LV remodeling in rats after MI. Table 1 compares the properties of various conductive biomaterials.

### 4.2. Piezoelectric Biomaterials in CVTE

**Figure 6:**
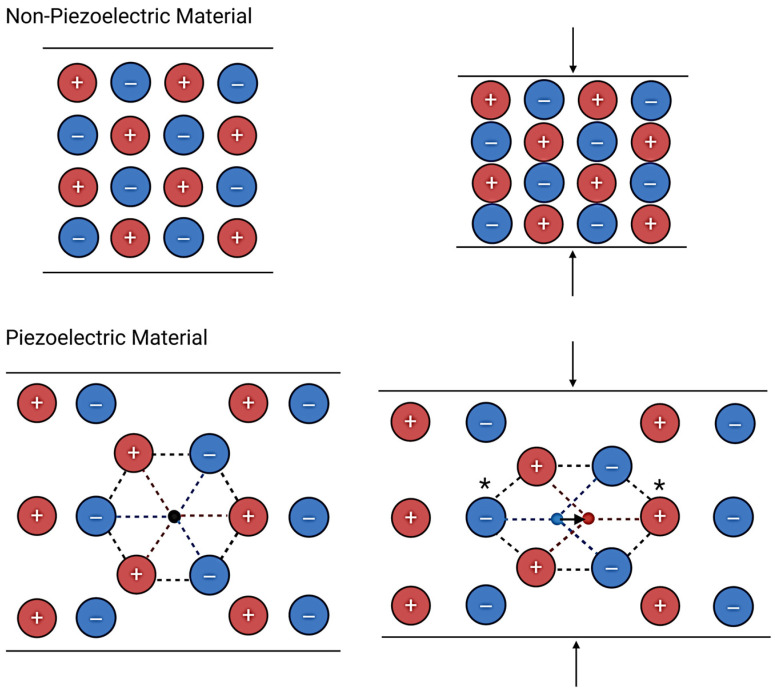
Mechanism of piezoelectricity. For a non-piezoelectric material, no dipole occurs even when deformation is applied. For a piezoelectric material, there is no net dipole without applied deformation, so the centroids of positive and negative charges are aligned at the black dot. When the material is deformed or compressed as depicted, the material elongates horizontally while the distance between atoms remains the same. The centroid of positive and negative charges is no longer aligned (red and blue dots) as the positive and negative charges separate (*), which creates a net dipole [159]. Image created with BioRender.com.

While conductive biomaterials passively restore endogenous signaling in CVTE, piezoelectric biomaterials actively generate exogenous electrical signals under mechanical deformation without needing a power source to modulate cell behavior and promote tissue regeneration. Harnessing the electro-responsiveness of cardiovascular cells will enable dynamic modulation of CVD progression. Electric fields produced by piezoelectric materials influence cells through similar mechanisms as external ES, including membrane potential, ion channels, and membrane receptors [36,38]. Piezoelectric materials are characterized by 2 opposing properties: (1) direct piezoelectric effect or the generation of electricity under applied force, and (2) inverse piezoelectric effect or material deformation in response to electrical input [38]. Piezoelectricity arises from the non-centrosymmetric structural arrangement of atoms, where mechanical deformation causes separation of positive and negative charges, creating a net electrical dipole, as depicted in Figure 6 [159]. In addition to physiological mechanical loading or external ultrasound acoustic stimulation, even subtle cell traction forces can activate piezoelectric biomaterials to generate in situ ES [160]. Piezoelectric performance is characterized by the piezoelectric coefficient d_xy_, which represents charge generated per unit of applied force or, conversely, deformation of the material per unit of applied voltage, with x and y indicating the directions of electric field and applied stress/strain, respectively. Commonly reported piezoelectric coefficients include longitudinal (d_33_), transverse (d_31_), and tangential (d_15_). Moreover, incorporating conductive materials can enhance piezoelectric performance [161,162,163]. Piezoelectric biomaterials have been widely explored in tissue engineering and hold strong potential for fabricating dynamic, responsive biomaterials and bioelectronic devices [36].

#### 4.2.1. Synthetic Piezopolymers

Common synthetic piezopolymers used in tissue engineering include polyvinylidene fluoride (PVDF), poly(L-lactic acid) (PLLA), and polyhydroxyalkanoates (PHAs). Polyvinylidene fluoride (PVDF) and its copolymer poly(vinylidene fluoride-trifluoroethylene) P(VDF-TrFE) have been widely used in biomedical applications due to their high piezoelectricity, processability, and flexibility [36]. Their piezoelectricity arises from its crystal phases (α, ꞵ, γ), with the ꞵ-phase exhibiting the highest piezoelectricity due to its all trans (TTTT) planar structure [164]. PVDF exhibits a piezoelectric constant (d_33_) of −30 pC/N, while P(VDF-TrFE), exhibiting higher flexibility, reaches −38 pC/N due to enhanced ꞵ-phase [36,165]. P(VDF-TrFE) has shown promise in CVTE. Adadi et al. reported the differentiation of hiPSC-CMs on P(VDF-TrFE) scaffolds, which also functioned as biosensors to detect contractile activity [166]. Monteiro et al. implanted P(VDF-TrFE)/PCL piezoelectric patches into infarcted mice and pig models, which improved electrical integrity, reduced ventricular dilation and hypertrophy, and downregulated ECM remodeling genes [167]. However, the insulating nature of PVDF and P(VDF-TrFE) limits their effectiveness for cardiac applications, which also require electrical conductivity to restore signal propagation over the damaged myocardium. Meira et al. enhanced conductivity of P(VDF-TrFE) films by incorporating 5% wt. graphene, which supported H9c2 rat cardiac myoblast adhesion and proliferation without signs of cytotoxicity [168]. The exhibited conductivities of 1.94 × 10^−3^ S/m (5% graphene) and 1.55 × 10^−1^ S/m (10% graphene), which was comparable to native myocardium (0.005–0.16 S/m). Similarly, Golafshan et al. showed bilayer graphene-modified PVDF nanofibers with pulsatile electrical stimulation (5 V/cm, 100 Hz, 20% duty ratio, 1 h/day) induced expression of cardiac marker α-actinin in H9c2 rat cardiac myoblasts [169]. Okoshi et al. in 1992 fabricated microporous small-diameter P(VDF-TrFE) vascular grafts (1.5 mm ID) using spray phase inversion and induced permanent dipoles via corona poling [170]. They hypothesized that the negative luminal surface and electrical charges generated by piezoelectric materials may reduce thrombosis and accelerate endothelialization. Although poled grafts initially generated more voltage than unpoled grafts, both showed similar patency rates (92% vs. 88%) and healing after 6 months, likely due to insufficient charge to exert a biological effect. Ma et al. designed a piezoelectric vascular graft for real-time hemodynamic monitoring of vascular health using PVDF nanofiber mats with AgNW electrodes sandwiched between two PCL layers [171]. However, PVDF-based materials suffer from poor biodegradability, limiting tissue integration [51]. Although exhibiting lower piezoelectricity, biodegradable piezoelectric polymers PLLA and PHBV are also being explored.

Polylactic acid (PLA) is an FDA-approved, biocompatible, biodegradable, and piezoelectric polymer derived from the fermentation of sugars and has been widely used in biomedical applications [172]. PLA, composed of D and L stereoisomers, can exist as semicrystalline PDLA and PLLA, or as amorphous PDLLA. Among them, poly(L-lactic acid) (PLLA) is the most studied for its piezoelectric properties, which arise from the displacement of C=O bonds [173,174]. PLLA exhibits several crystalline forms (α, α′, ꞵ, γ), with the α form being the most stable and the ꞵ form being the most piezoelectric. PLLA exhibits a shear piezoelectric coefficient (d_14_) of 6–12 pC/N and a normal piezoelectric coefficient (d_33_) of 3.08 pC/N [172]. Various processing techniques can enhance the piezoelectricity of PLLA. For example, mechanical stretching at a draw ratio of ~5 aligns dipoles and increases d_14_ to 11.25 pC/N [175], while electrospinning under high voltage stretches randomly oriented chains (α-form) into aligned chains (ꞵ-form), enhancing piezoelectricity [173,175]. Increasing collector drum speed also increases fiber alignment and crystallinity, boosting d_14_ to 19 pC/N and charge output to 0.9 nC at 4000 rpm [176]. Piezoresponse force microscopy (PFM) studies showed that decreasing nanofiber diameter (especially under 100 nm) exponentially increased d_33_ [177]. Additionally, heat treatment (65 °C) near the glass transition temperature (66 °C) also modulates PLLA voltage output, increasing longitudinal output (up to a threshold temperature), but reducing transverse output. Although PLLA has been extensively used in CVTE for cardiac patches and vascular grafts [178,179,180], its piezoelectric properties have been less explored in this context. Zhao et al. developed an electrospun biodegradable piezoelectric PLLA scaffold coupled with ultrasound to deliver ES for MI repair [181]. This wireless system enhanced mitochondrial function, angiogenesis, and regulated intracellular Ca^2+^ concentration in CMs as well as contraction rhythm in heart tissues, offering an innovative ES platform for heart disease treatment. To overcome limitations of PLLA, such as hydrophobicity, brittleness, low piezoelectricity, and inflammatory lactic acid byproducts, blending with other polymers can improve cell adhesion and tissue regeneration [174]. Incorporating low molecular weight PCL (Mw = 2000 Da, 5%) or poly(glycerol sebacate) (PGS) (30%) enhances hydrophilicity, mechanical properties, and surface functionalization of PLA-based scaffolds [179,182]. Furthermore, piezoceramic nanoparticles, which will be discussed later, can also be incorporated to enhance piezoelectricity [183,184]. Wang et al. developed an ultrasound-responsive Li-doped ZnO/PLLA piezoelectric scaffold that eliminated bacteria under sonodynamic therapy, reduced inflammation, and promoted healing through vascularization [185].

Polyhydroxyalkanoates (PHAs), including poly(3-hydroxybutyrate) (PHB) and its copolymer poly(3-hydroxybutyrate-3-hydroxyvalerate) (PHBV), are another class of biodegradable piezopolymers derived from bacteria as a form of carbon source and energy storage [38]. PHBV, which incorporates monomers such as valeric, exhibits a piezoelectric coefficient of 1.3 pC/N and offers greater flexibility and elasticity than PHB [184,186]. It also degrades more slowly than other polymers through enzymatic action and hydrolysis, releasing CO_2_. Although PHAs have low piezoelectric coefficients (1.6–2.0 pC/N), they exhibit suitable biodegradability, biocompatibility, and mechanical properties for scaffolds and biosensors [38]. In cardiac repair, Castellano et al. showed that electrospun PHB scaffolds outperformed PCL, silk, PLA, polyamide, and collagen by simultaneously promoting adhesion of MSCs, CMs, and cardiac fibroblasts, although lower than PCL [187]. The scaffolds also degraded by 8 weeks in vivo, demonstrated graft tolerance (upregulated IL-6 and IL-10 expression in peripheral blood mononuclear cells), increased angiogenesis, and promoted M2 macrophage polarization in rat heart models. Majid et al. utilized melt electrowriting to fabricate high molecular weight, medium chain-length PHA (MCL-PHA) cardiac patches, supporting hPSC-CMs maturation in vitro [188]. Incorporation of ECs enhanced vascularization in a murine MI model without fibrous capsule formation, although the patches failed to retain the seeded hPSC-CMs or prevent cardiac function decline. To enhance piezoelectricity for clinical applications, PHB/PHBV often incorporates inorganic fillers such as zinc oxide (ZnO) or rGO [186]. Zviagen et al. hydrothermally deposited ZnO onto PHB scaffolds, which enhanced d_33_ from 2.9 ± 0.1 pC/N to 13.7 ± 1.6 pC/N, increased electrical output from 0.58 ± 0.02 V to 0.88 ± 0.04 V, and improved wettability [189]. Toala et al. blended chitosan with 13% PHB in acetic acid to form thin films, achieving an apparent d_33_ of ~200 pC/N due to synergistic effects of piezoelectricity and electrostriction [190]. Although numerous studies have utilized PHAs in tissue engineering, the specific application of their piezoelectric properties in CVTE remains largely unexplored [191].

#### 4.2.2. Natural Piezoelectric Biomaterials

Many natural biomaterials also exhibit inherent piezoelectricity, including proteins such as collagen, elastin, and silk fibroin; polysaccharides such as chitin, chitosan, and cellulose; and biomolecules such as amino acids and peptides [19,192]. While they typically have lower piezoelectric coefficients, natural piezoelectric biomaterials offer key advantages, including high biocompatibility, biodegradability, and low toxicity, making them attractive for tissue engineering applications [38,51].

Collagen, the most abundant protein in mammalian tissues, is composed of a triple helix structure formed by three polypeptide chains with a repeating Gly-X-Y sequence, where X and Y are typically proline and hydroxyproline [19,193]. Reported piezoelectric coefficients include ~2 pm/V in collagen fibrils, 0.44 pm/V in collagen films, and 12 pm/V (d_14_) in rat tail collagen [51,192]. Elastin, another ECM protein found in elastic tissues such as blood vessels, lungs, and skin, exhibits lower piezoelectricity (~1 pm/V in aortic elastin and 0.1 pm/V in murine lung) [192]. Unlike collagen, it also displays ferroelectricity, which enables electrical poling to enhance its piezoelectric strength. Silk, composed of fibroin and sericin, is extracted from silkworms and demonstrates high biocompatibility, tunability, flexibility, and excellent mechanical properties [192]. By removing sericin during the degumming process, pure silk fibroin can be obtained, exhibiting higher mechanical strength and a d_33_ of 38 pm/V and d_14_ of 1.5 pC/N [19,192]. Chitin is a biodegradable polysaccharide derived from exoskeletons of crustaceans and insects, exhibiting a piezoelectric coefficient of ~0.2–1.5 pC/N, with d_33_ reaching 9.49 pC/N in nanofibers [38,194]. Its n-deacetylated derivative, chitosan, shows improved solubility and higher piezoelectricity, with reported d_33_ values of 18.4 pC/N in particles and 15.56 pC/N in films [19]. Cellulose, composed of a linear homopolymer of glucose, is another common polysaccharide with a low piezoelectric coefficient of ~0.1 pC/N [38]. However, different processing techniques can enhance its piezoelectricity. Cellulose nanocrystal (CNC) films can reach a d_33_ of 29 pC/N, while cellulose nanofiber (CNF)-based generator can achieve a d_33_ of 46 pm/V [19]. An emerging piezoelectric biomaterial is eggshell membrane (ESM), a low-cost, biocompatible, and biodegradable kitchen waste byproduct rich in ECM proteins such as collagen, glycosaminoglycans, and fibronectin [195]. ESM exhibits a d_33_ of 23.7 pC/N and has been utilized in energy harvesters, electronics, biosensors, wound healing, nerve regeneration, and other tissue engineering applications. Although many of these natural biomaterials have been extensively used for CVTE, most studies focused on their biochemical and mechanical characteristics rather than leveraging their electrical properties [76,196,197,198,199,200,201].

Although less commonly discussed in tissue engineering, protein building blocks such as amino acids like glycine and peptides like diphenylalanine (FF) also exhibit useful piezoelectric properties for energy harvesting. For example, β- and γ-glycine amino acid crystals exhibit piezoelectric coefficients of 178 pm/V (d_16_) and 10 pm/V (d_33_), respectively [175]. The high d_16_ of β-glycine results from efficient molecular packing, although its instability (easily converts into α and γ forms) and water solubility limit its biomedical applications unless encapsulated in water-resistant polymers [19,175]. Chorsi et al. incorporated glycine into electrospun PCL nanofibers to fabricate an ultrasound transducer for drug delivery, achieving a d_33_ of 19 pC/N [202]. FF, a peptide formed by two phenylalanine molecules, exhibits high piezoelectricity, flexibility, biocompatibility, and mechanical strength [175]. FF can be formed into various micro and nano structures, such as aligned nanotubes, which demonstrated a d_15,eff_ of 67.76 pm/V. The asymmetry of FF nanotubes enables efficient conversion of shear deformation into electrical energy [19]. However, similar to glycine, FF also suffers from in vivo instability due to moisture susceptibility and rapid degradation [175]. While the piezoelectricity of natural biomaterials is generally low compared to synthetic polymers, they can be modified through fabrication techniques or functionalized with other piezoelectric or conductive compounds to enhance their electrical properties and stability [38,192].

#### 4.2.3. Piezoelectric Bioceramics

Piezoelectric bioceramics possess significantly higher piezoelectricity than natural and synthetic piezoelectric materials, but are very brittle and cannot be used directly for tissue engineering [174]. Therefore, they are often combined with polymers, which enhances the polymer’s piezoelectricity while maintaining high processability. The piezoelectric properties can be tuned via electric poling or temperature; however, high temperatures above the Curie point compromise piezoelectricity. Some piezoceramics that have been used in CVTE include barium titanate (BaTiO_3_) and zinc oxide (ZnO), which have the advantages of low cost, acceptable biocompatibility, and chemical stability [186].

BaTiO_3_ is a perovskite piezoceramic that exhibits strong piezoelectricity (d_33_ = 191 pC/N) in its tetragonal phase [174]. It demonstrates high biocompatibility even at high concentrations and has been widely used as nanoparticles in tissue engineering. Cafarelli et al. incorporated BaTiO_3_ nanoparticles (~300 nm) into small-diameter poly(ether)urethane (PEtU)/polydimethilsyloxane (PDMS) vascular grafts, achieving a d_33_ of 1.91 pm/V [203]. While the biological effects of piezoelectric stimulation were not explored, the doped grafts showed comparable mechanical properties to controls, higher burst strength (~1100 kPa vs. ~800 kPa), and long-term stability (2% mass loss over 6 months). Compositing piezoelectric and conductive biomaterials, Liu et al. recently developed a multilayer scaffold composed of eggshell membrane (ESM), polyethylene oxide/polyaniline (PEO@PANI), and polylactic acid/BaTiO_3_ (PLA@BT) to promote rapid EC proliferation [204]. The scaffold achieved a d_33_ of up to 82.4 pC/N, generated a voltage output to 1.019 V, and promoted HUVEC adhesion, proliferation, and alignment. Upon ultrasonic stimulation (0.1 W/cm^2^), HUVEC proliferation increased by 257.29% compared to unstimulated controls, highlighting its potential for vascular tissue engineering.

ZnO is a wurtzite piezoceramic with an asymmetric hexagonal structure and a d_33_ of 6–13 pC/N [172,174]. It has robust antibacterial activity, ROS scavenging potential, and the ability to promote cell proliferation, making it attractive for nerve regeneration and wound healing [186]. Augustine et al. incorporated ZnO nanoparticles (up to 4% *w*/*w*) into electrospun P(VDF-TrFE) scaffolds to enhance cell adhesion and angiogenesis [205]. The scaffolds demonstrated good hemocompatibility with RBCs, WBCs, and platelets, and improved MSC and HUVEC adhesion and proliferation. Implantation into rats showed improved angiogenesis after 21 days, likely due to a combination of piezoelectric effect and ROS generation (O_2_^−^/H_2_O_2_), which stimulated VEGF and FGF production. While ZnO above micron size has not shown toxicity, concerns remain for ZnO nanoparticles due to ROS production [184]. Mechanisms of ZnO-induced toxicity include oxidative stress, Zn^2+^ release and accumulation, autophagy induction, and ion channel disruption such as Ca^2+^ channels [206]. There are similar cytotoxicity concerns about BaTiO_3_ nanoparticles at high concentrations, where a concentration of 100 µg/mL induced significant cytotoxicity in Rhesus monkey endothelial cells (RhRECs), although a lower concentration at 10 µg/mL showed viability above 90% [207]. Overall, the cytotoxicity of piezoceramics is influenced by their dosage, particle size, and exposure duration. Further research is necessary to fully investigate their long-term biosafety for use in tissue-engineered scaffolds.

Overall, synthetic and bioceramic piezoelectric biomaterials demonstrate sufficient piezoelectric responses under external ultrasound stimulation to produce physiologically relevant electrical cues, whereas natural biomaterials are generally used for their biological properties, apart from piezoelectric amino acids and peptides. Physiological mechanical stimuli such as cardiac mechanical loading and pulsatile blood flow have also been shown to produce sufficient piezoelectric responses for therapeutic ES or energy harvesting. For example, piezoelectric cardiac patches producing approximately 50–60 mV under cyclic heart movement were able to attenuate ventricular remodeling in infarcted mice hearts [167]. Piezoelectric vascular grafts also enabled self-powered real-time hemodynamics monitoring of pulsatile blood flow [171]. While many studies still utilize external ultrasound to induce sufficient piezoelectric output, the field is moving towards self-powered bioelectronic systems that are adaptive to the biological environment [97]. Table 2 compares the properties of various piezoelectric biomaterials.

### 4.3. Other Electroactive Biomaterials

In addition to conductive and piezoelectric materials, other classes of electroactive materials, such as pyroelectric, ferroelectric, triboelectric, and magnetoelectric, can also generate or respond to electrical signals through different mechanisms. Piezoelectric materials constitute a broad class of electroactive materials, within which pyroelectrics are a subset, and ferroelectrics are in turn a subset of pyroelectrics [216].

Pyroelectric materials, a subclass of piezoelectric materials, generate electric fields in response to temperature changes, making them useful for body heat-powered implants, thermometry, infrared sensing, and medical imaging [217]. Temperature fluctuations lead to the separation of positive and negative charges, inducing changes in polarization and the generation of an electric field. Some examples include BaTiO_3_, Bi_13_S_18_I_2_, and NaNbO_3_. Yu et al. developed a photothermal-pyroelectric biosensor to diagnose acute myocardial infarction by detecting cTnI protein from blood [218]. After capturing the biomarker using immobilized antibodies and illuminating the photothermal probes with an NIR laser, the heat signal generated was efficiently converted into detectable electrical signals using pyroelectric NaNbO_3_ microelectrodes, enabling rapid detection (<1 h) and high sensitivity (50 pg/mL). Likewise, thermoelectric materials like Bi_2_Te_3_ convert temperature gradients into electric current, with electrons flowing from the hot to the cold end of the material. Although these materials have been utilized in biosensors and wearable electronics, the application of their pyroelectric effect in tissue engineering remains limited.

Ferroelectric materials, a subclass of pyroelectric materials and therefore piezoelectric, exhibit intrinsic spontaneous electrical polarization that can be reoriented by an external electric field [219]. Several types of ferroelectric biomaterials include PVDF, BaTiO_3_, KNN, and BiFeO_3_, and organic/inorganic hybrids [217]. Ferroelectric materials have been utilized for sensors, biological catalysis, and energy harvesting in triboelectric nanogenerators (TENGs) [217,219]. Li et al. developed a ferroelectric artificial artery using PVDF and potassium sodium niobite (KNN) particles for battery-free blood pressure and occlusion monitoring [220]. Sekine et al. printed a wearable ferroelectric PVDF-TrFE sensor to monitor pulse rate from blood flow [221]. However, these materials are mostly used for their piezoelectric properties in cardiovascular applications, as previously described.

Triboelectric materials generate electricity through contact electrification, where friction between two dissimilar materials coming into contact and then separating leads to electron transfer from one material to the other, creating oppositely charged surfaces [222]. The resulting potential difference powers TENGs, which have been applied to various cardiovascular systems such as driving pacemakers without the need for battery replacement, powering biosensors, or generating ES to promote CM maturation for myocardial repair [223]. Tufan et al. developed silk fibroin/carbon nanofiber TENG electrodes that promoted iPSC differentiation into CMs under simulated cardiac motion in vitro and upregulated cardiac markers (TNNT2, NKX2.5) [224]. The scaffolds exhibited a conductivity of 0.021 ± 0.006 S/cm and generated a power output of 0.37 × 10^−3^ mW/m^2^, open-circuit voltage of 0.46 V, and short circuit current of 4.5 nA. Qiu et al. engineered a TRI-TENG integrating rGO and PDA/rGO electrodes, patterned PVDF, and PEDOT:PSS/GelMA hydrogel, which can harvest the heart’s mechanical energy, generate ES, facilitate electrical signal propagation, and serve as a potential biosensor for diagnosis [225]. The device improved cardiac function in MI pigs by 14.7% after 28 days and modulated mRNA expression related to cardiac muscle contraction, energy metabolism, and vascular regulation in rat hearts.

Magnetoelectric materials experience electric polarization under a magnetic field (direct effect) or magnetization under an electric field (converse effect) [226]. Recent advancements have led to the development of room-temperature magnetoelectric composites by combining piezoelectric and ferromagnetic phases, which is a significant breakthrough for biomedical applications. In these systems, an external magnetic field induces mechanical deformation of the ferromagnetic phase via magnetostriction, which in turn activates the piezoelectric phase to generate an electric field. Some magnetoelectric composites for tissue engineering include Fe-doped hydroxyapatite, cobalt ferrite (CoFe_2_O_4_), Terfenol-D, Fe_3_O_4_, and Metglas mixed with polymers such as PLLA, silk fibroin, or P(VDF-TrFE). Jing et al. developed a magnetostrictive CoFe_2_O_4_-PVDF cardiac patch that improved the proliferation, spreading, and contraction of oxidative stress-injured H9c2 rat cardiac myoblasts via upregulation of steroid hormone biosynthesis, Na^+^/K^+^-ATPase, and intracellular Ca^2+^ [227].

The ability of these electroactive materials to harvest energy from physiological activities has opened new avenues for the next generation of self-powered cardiovascular monitoring and intervention therapy devices, including blood pressure sensors, vascular graft sensors, pacemakers, and cardiac patches [228]. While extensive research is still needed before clinical translation, the unique electrical properties of these biomaterials show great promise to advance biomedical technologies and improve human health.

## 5. Current Challenges and Future Directions of Electroactive Biomaterials for CVTE

Electroactive biomaterials show high potential in tissue regeneration and have garnered significant interest for CVTE. However, several challenges remain, including tissue-specific variability, optimization of material properties, biodegradability, and long-term safety [33,53]. A major challenge lies in understanding tissue-specific responses to ES when designing electroactive biomaterials [33]. Tissues are heterogenous structures, comprising diverse cell types with distinct responses to ES. For instance, in vascular grafts, different ES parameters can modulate platelet activity, enhance endothelialization, and influence VSMC phenotype [27,87,93]. Modulating the behavior of one cell type may inadvertently harm another. The underlying mechanisms of how electrical cues influence specific cell behavior remain poorly understood, and further research is needed to elucidate the role of endogenous bioelectricity in regulating differentiation, proliferation, and migration for targeted ES.

To achieve targeted regeneration, electroactive biomaterials must be engineered to mimic the native bioelectrical environment [53]. Designing scaffolds with spatially distributed properties can better match the heterogeneity of the native tissues. Additionally, other essential properties, such as mechanical strength, must be considered to prevent failure [33]. Current research often prioritizes maximizing the electrical output of piezoelectric scaffolds, neglecting cell-specific requirements for effective ES [186]. Variations in cell culture platforms also complicate the standardization of ES parameters, as identical parameters can yield vastly different outcomes [36]. Moreover, the integration of external ES with conductive scaffolds further demands precise optimization of parameters, such as voltage, frequency, and duty cycle, specific to each cell or tissue type [33]. To facilitate the clinical translation of ES, standardized protocols for reporting must be established [229]. First, ES parameters should be reported, including waveform, electric field strength, frequency, duty cycle, pulse width, and stimulation duration. Second, standardized in vitro and in vivo testing platforms should include report material and electrode types, setup configurations, cell types, culture conditions, animal models, and experimental procedures. Lastly, a unified evaluation of biological effects from ES should be established, such as inflammation resolution, CM survival, and angiogenesis. These standardized processes will help optimize ES parameters and establish benchmarks for comparison across studies.

Another critical concern is long-term safety during biomaterial degradation. The degradation rate must align with tissue regeneration without degrading too rapidly or too slowly, disrupting the repair process [53]. Degradation may also alter the electrical properties of the biomaterial, potentially impairing regeneration. Some materials, such as PVDF and PLLA, show significantly reduced piezoelectricity under extended cyclic loading [186]. Others, particularly conductive materials such as carbon-based elements, can leach toxic compounds as the scaffold degrades [33], while piezoceramic nanoparticles such as BaTiO_3_ and ZnO have been shown to generate ROS and induce oxidative damage [206]. Utilizing computer-aided design and simulation can help predict material degradation and long-term performance [53]. Furthermore, many high-performance piezoelectric materials, such as BaTiO_3_ and PVDF, are also not biodegradable, requiring blending with biodegradable polymers to enhance biodegradability and reduce the risk of chronic inflammation. Comprehensive studies on immune response, cytotoxicity, and inflammation are essential before clinical translation, and further research will need to balance biocompatibility, biodegradability, and stable electrical performance.

To advance the use of electroactive biomaterials in tissue engineering, future studies must address the limited understanding of cell response to electrical stimuli, which is essential for effectively guiding cell behavior. This requires the development of more reliable and complex in vitro models [33]. Precisely recapitulating the target microenvironment can improve assessments of efficacy and safety prior to in vivo studies. Cardiovascular organ-on-a-chip platforms, such as heart-on-a-chip and vasculature-on-a-chip platforms, are next-generation in vitro models that can mimic the human in vivo microenvironment, incorporate biochemical, biomechanical, and electrical cues, and accelerate clinical translation of therapies [230]. Ren et al. developed a heart-on-a-chip platform capable of electrical stimulation to promote maturation of CMs for drug screening [231]. In addition to mechanistic studies, cardiovascular prosthesis can also be evaluated in these platforms. Bot et al. developed a microscale vascular graft-on-a-chip to evaluate thrombogenicity under varying flow conditions [232]. These studies highlight the potential of organ-on-a-chip platforms to bridge electroactive materials and devices to clinical translation. Beyond tissue engineering, electroactive biomaterials, specifically piezoelectric materials, have significant potential for self-powered smart bioelectronics as they can generate electricity. They can be used as biosensors to detect weak biological signals, supporting real-time health monitoring and personalized medicine. With the recent advancements in flexible electronics, miniaturization, and nanotechnology, piezoelectric biomaterials can revolutionize biosensor technology for cardiovascular implants, enabling early intervention and shifting healthcare from a reactive to a proactive system [5,53].

The regulatory pathway for marketing cardiovascular bioelectronic implants requires stringent assessment to ensure the safety and efficacy of the medical device, which is overseen by the Food and Drug Administration (FDA) in the United States. The medical device life cycle follows the general framework: (1) concept and design, (2) preclinical testing, (3) clinical evaluation, (4) regulatory approval, and (5) post-market surveillance via the FDA’s Medical Device Reporting (MDR) system to ensure long-term safety [233]. Implantable vascular devices are generally classified as class III (high risk) due to their invasiveness, requiring a comprehensive assessment of biocompatibility, durability, safety, and long-term performance. Extensive evaluation of biological responses to the implant, including inflammation, thrombosis, and fibrous capsule formation, is essential to ensure the implant’s effectiveness and longevity, compliance with safety regulations, and successful clinical translation.

## 6. Conclusions

CVD remains the leading cause of death worldwide, highlighting the urgent need for advanced biomaterials capable of effectively restoring cardiovascular tissues. Electroactive biomaterials, which integrate electrical, mechanical, and biochemical cues, offer significant potential to modulate CVD progression. Among these, conductive biomaterials have been used extensively in cardiac applications to restore electrical signal propagation over the damaged myocardium and are more clinically mature due to their clearer mechanistic understanding and simpler fabrication. CNTs and gold are two of the most used conductive biomaterials in cardiac tissue engineering [234]. In contrast, piezoelectric biomaterials, which generate electrical cues to modulate cellular responses, are emerging as a promising therapeutic strategy in CVD treatment but are more mechanistically complex and require further validation. PVDF remains the most widely used material for piezoelectric stimulation due to its strong piezoelectric properties [229]. Notably, both platforms can be integrated to synergistically enhance the efficacy of ES for tissue regeneration [53]. The current landscape of electroactive materials often relies on external power sources for electrical stimulation or mechanical activation of the piezoelectric effect via ultrasound, thereby limiting clinical translation and requiring patient compliance. The next generation of electroactive biomaterials and devices should harness endogenous physiological cues to generate electrical energy for self-powered bioelectronics. More importantly, future development should move beyond maximizing electrical properties alone or targeting a single cell population, and instead tailor designs that adapt to the dynamic pathophysiological environment. Given the numerous cellular interactions within a disease environment, a key design consideration is how these materials can regulate cellular crosstalk to promote functional tissue repair. Furthermore, the materials should be tailored to different stages of the tissue repair process, focusing on inflammation modulation during the acute response stage, followed by angiogenesis for subsequent tissue regeneration. Although several critical challenges remain, including incomplete understanding of cellular responses to electroactive signals, the need for precise and controlled electrical stimulation to guide cell behavior, and concerns regarding long-term safety, electroactive biomaterials represent a versatile platform for dynamically regulating CVD and enabling real-time health monitoring, laying the groundwork for the next generation of smart cardiovascular devices.

## Figures and Tables

**Table 1:** Comparison of conductive biomaterials.

Conductive Material Classification	Material	Conductivity	Pros	Cons	References
Metal	Au (bulk, NPs)	4.11 × 10^5^ S/cm (bulk), 86 S/cm (4 nm NP), 8.3 S/cm (2 nm NP)	High biocompatibility, high conductivity, oxidation resistance, ease of fabrication	Size and shape-dependent toxicity, smaller sizes have greater cytotoxicity	[36,101,102]
	AuNP (*S. aureus*-derived), Dopamine/ GelMA/PEGDA/AuNP (DPGA)	3.282 × 10^−6^ ± 8 × 10^−7^ S/cm	High bioactivity, promoted CM function, improved MI repair	Require purification step to remove toxic components from *S. aureus*, low conductivity	[106]
	AuNP/ECM/silk fibroin	~1.5 × 10^−3^ S/cm	Promoted cardiac repair and decreased infarct size from 89% to 65%	Patches seeded with MSCs have limited impact due to low cell retention	[107]
	Ag (bulk)	6.30 × 10^5^ S/cm	High conductivity, antimicrobial properties	Cardiotoxicity due to inhibition of ion channel activity	[101,112,114]
	AgNP/collagen	8 × 10^−9^ S/cm	Enhanced rat neonatal rat ventricular myocyte proliferation and Cx43 expression, inhibited biofilm formation, and did not activate macrophages	Require external electrical stimulation	[111]
	MXene (pure film)	2.0–2.4 × 10^4^ S/cm	High conductivity, hydrophilicity, and biocompatibility	Unclear long-term safety, potential toxicity, in vivo oxidation and instability, challenging scalability	[116,117]
	MXene nanosheet (Ti_3_C_2_T_x_)/PVA/PAM	4.11 × 10^−2^ S/cm	High toughness, conductivity, biocompatibility, reduced CM apoptosis by 15.83% under hypoxia	Degradation, oxidation, and loss of conductivity after 12 days (60 °C) or 16 days (37 °C)	[119]
	MXene nanosheet (Ti_3_C_2_)/silk fibroin/hyaluronic acid	3.2 × 10^−3^ S/cm	Improved LVEF and LVFS in MI rats	High degradation (19% mass loss after 28 days in PBS)	[120]
Carbon	Graphene nanosheet and carbon nanotube (CNT)	10^6^ S/cm (graphene), 10^4^–10^5^ S/cm (CNT)	High conductivity, chemical stability, large surface area	Hydrophobicity, minimal biodegradation, varying toxicity depending on size, shape, fabrication	[123,133,158]
	rGO nanolayer/silk (anisotropic conductivity using aligned and random fibers)	0.2–0.3 S/cm	Improved myocardial repair, pumping function, CM survival, and angiogenesis	Not suture-free, unclear inflammatory response and cytotoxicity, further optimization of mechanical properties	[125]
	TEG-graphene (nanoparticle)/ GelDA	6.24 × 10^−3^ S/cm	High conductivity matching native myocardium, strong mechanical properties, consistent degradation, suture-free, improved cardiac function in MI rats	Short in vivo assessment for 28 days, but long-term effects are unknown	[126]
	Sulfonated carbon nanotubes (SCNT)/(TEMPO)-oxidized cellulose nanofibrils (TOCN)	8.4 × 10^−4^ − 6.2 × 10^−2^ S/cm	Low % of CNT, high biocompatibility, increased Cx43 expression	No in vivo validation	[130]
	CNF (nanofiber) /gelatin	8.39 × 10^−7^ S/cm	Enhanced cardiac gene expression (TrpT-2, ACTN4, Cx43) and angiogenic potential	Further research into myocardial repair and degradation/release of CNFs in vivo	[132]
Conductive polymer	PPy	10^2^–7.5 × 10^3^ S/cm	Good biocompatibility, stimuli-responsive, high conductivity, good chemical stability	Low processability, non-thermoplastic, brittle	[134]
	PPy/silk fibroin	11.22 ± 0.63 × 10^−4^ S/cm	Similar conductivity as native myocardium, enhanced cardiac markers expression, good mechanical properties, improved cardiac functions in vivo	Require external electrical stimulation, implanted patch not cell-free	[136]
	PANI	30–200 S/cm	High conductivity, ease of synthesis, good stability, ability to switch conductive/resistive states	Low processability, low flexibility, non-biodegradability, risk of chronic inflammation	[134]
	f-PANI/PVA	0.0105–0.0138 S/cm	Simultaneous cardiac monitoring and electrocoupling therapy, suture-free	Connects to external device for monitoring, long-term safety and efficacy testing required, scale from rat to pig models	[141]
	PANI/PU	4.66 × 10^−2^ S/cm	Support HUVEC proliferation and exhibit anti-thrombotic effects	No in vivo validation	[143]
	PEDOT:PSS	1–4700 S/cm	Biocompatibility, good conductivity, water solubility, versatility	Additional processing steps required, optimization of long-term safety, conductivity, and stability	[36,144,145,146]
	PEDOT:PSS/collagen/alginate	35 ± 11 × 10^−4^ S/cm	Enhanced CM maturation and synchronous beating without external mechanical or electrical stimulation	Cytotoxicity at higher PEDOT:PSS concentration (0.52 *w*/*w*%) in 3D	[148]
	PEDOT:PSS/ PVA/ N-cadherin peptide	~40 S/cm	No cytotoxicity to cardiac fibroblasts, high elasticity, antibacterial properties	Need to test wider range of bacteria and effect of other sterilization methods on hydrogel properties	[149]
	PEDOT:PSS/ GelMA	1.121 × 10^−3^ S/cm (G15P0.3)	Injectable, good biocompatibility, hemocompatibility, elastic modulus, and electrochemical properties, improved cardiac function in MI rabbit model	Require UV crosslinking, complex fabrication of OECT device, in vivo testing only for hydrogel and not complete device	[151]
Metalloid	SiNW (nanowire)	1.50–5.00 S/cm	Enhanced hiPSC-cardiac spheroid maturation	Required exogenous electrical stimulation (2.5 V/cm, 1 Hz, 5 ms, day 10–19)	[152]
Ionic conductive biomaterials	Chitosan/lipoic acid/proanthocyanidins/Eu^3+^ (CS/TA@PAs-Eu)	1.3 × 10^−4^ S/cm	Improved mitochondrial function, ROS scavenging, and angiogenesis and restored cardiac function in rats	Hydrogels require suturing to the heart	[157]

**Table 2:** Comparison of piezoelectric biomaterials.

Piezoelectric Material Classification	Material	Piezoelectric Coefficient	Pros	Cons	References
Synthetic	P(VDF-TrFE)	d_33_ = −38 pC/N, d_31_ = −23 pC/N	High piezoelectricity and biocompatibility	Poor biodegradability, hydrophobicity	[38,208]
	PLLA	d_33_ = 3.08 pC/N, d_14_ = 6–12 pC/N	High biocompatibility and biodegradability	Low piezoelectricity, hydrophobicity	[172]
	PHA/PHB/PHBV	1.6–2.0 pC/N	Biodegradable, biocompatible, and suitable mechanical properties	Low piezoelectricity, hydrophobicity	[38,189]
Natural	Collagen	2 pm/V, d_14_ = 12 pm/V	Biocompatible, biodegradable, FDA-approved, commercially available	Low piezoelectricity and mechanical strength, risk of disease transmission, variable properties depending on extraction process	[192,209]
	Elastin	1 pm/V	Biocompatible, biodegradable, imparts material elasticity, promotes EC proliferation, anti-thrombogenic, and anti-inflammatory properties	Low piezoelectricity, insolubility, not suitable for mass production	[192,210,211]
	Silk fibroin	d_33_ = 38 pm/V, d_14_ = 1.5 pC/N	Biodegradable, flexible, high mechanical strength, low immunogenicity	Low bioactivity, heterologous origin, difficult to preserve and transport	[19,192,211]
	Chitin	0.2–1.5 pC/N, d_33_ = 9.49 pC/N (nanofiber)	Widely available from crustaceans and insects, hydrophilic, promotes cell adhesion and spreading	Poor processability, insoluble in water and most organic solvents, limited large-scale use	[38,212,213]
	Chitosan	18.4 pC/N	Low cost, antimicrobial, anti-thrombogenic, bioactive, hydrophilic, similar structure as GAG proteins in ECM	Poor mechanical properties	[211,214]
	Cellulose	0.1 pC/N, d_33_ = 46 pm/V (CNF film), d_33_ = 29 pC/N (CNC film)	Low cost, easily sourced, biocompatible, bioactive, tunable mechanical properties	Not degradable in humans	[19,38,215]
	Eggshell membrane	d_33_ = 23.7 pC/N	Biodegradable, low cost, readily available, diverse applications in bioelectronics and tissue regeneration	Batch-to-batch variation, complex extraction and purification, limited solubility due to crosslinked disulfide bonds	[195]
	Glycine (β/γ)	d_16_ = 178 pm/V (β), d_33_ = 10 pm/V (γ)	Biocompatible, high piezoelectricity	Low stability, water soluble	[19,175]
	Diphenylalanine (FF)	d_15, eff_ = 67.76 pm/V	High piezoelectricity, flexible, biocompatible, high mechanical strength	Low stability, moisture susceptibility, rapid in vivo degradation	[175]
Bioceramics	BaTiO_3_	d_33_ = 191 pC/N, d_31_ = −78 pC/N	High piezoelectricity, good biocompatibility even at high concentrations	Non-biodegradable, potential toxicity and oxidative damage	[172,206]
	ZnO	d_33_ = 6–13 pC/N, d_31_ = −5 pC/N	Biocompatible, biodegradable, antibacterial	Potential toxicity to respiratory, digestive, nervous, urinary, and hematopoietic systems from ROS, Zn^2+^ accumulation, ion channel disruption	[172,206]
Composites	P(VDF-TrFE)/PCL	d_33_ = 14.7 ± 0.4 pm/V	Cardiac patch improved electrical integrity, decreased ECM remodeling genes, reduced LV dilation and hypertrophy	No implantable ECG device for continuous monitoring of abnormal beating due to technical and ethical limitations; further studies needed on in vivo degradation and long-term effects on heart function	[167]
	PHB/ZnO	d_33_ = 13.7 pC/N	Enhanced piezoelectric response, output surface electrical potential, and wettability	No cytotoxicity studies	[189]
	Chitosan/PHB	d_33_ = 200 pC/N (apparent)	High piezoelectric coefficient	No cell culture studies	[190]
	ESM-PEO@PANI-(O)PLA@20%BT	d_33_ = 80.6 pC/N, d_31_ = 2.36 pC/N	High piezoelectric coefficient and suitable mechanical properties; promoted EC alignment and proliferation by 257.29% under ultrasound stimulation	Complex 3-layer fabrication process	[204]

## Data Availability

No new data were created or analyzed in this study. Data sharing is not applicable to this article.

## Abbreviations

The following abbreviations are used in this manuscript:

AC	Alternating current
AgNP	Silver nanoparticle
AuNP	Gold nanoparticle
AVN	Atrioventricular node
CM	Cardiomyocyte
CNF	Carbon nanofiber
CNT	Carbon nanotube
CVD	Cardiovascular disease
CVTE	Cardiovascular tissue engineering
DC	Direct current
EC	Endothelial cell
ECM	Extracellular matrix
ES	Electrical stimulation
ESM	Eggshell membrane
GelDA	Dopamine-modified gelatin
GelMA	Gelatin methacrylate
hiPSC	Human induced pluripotent stem cell
HUVEC	Human umbilical vein endothelial cell
MI	Myocardial infarction
MMP	Matrix metalloproteinase
MSC	Mesenchymal stem cell
PANI	Polyaniline
PCL	Polycaprolactone
PEDOT:PSS	Poly(3,4-ethylene dioxythiophene) polystyrene sulfonate
PHA	Polyhydroxyalkanoate
PHB	poly(3-hydroxybutyrate)
PHBV	poly(3-hydroxybutyrate-3-hydroxyvalerate)
PLLA	Poly(L-lactic acid)
PPy	Polypyrrole
PVA	Polyvinyl alcohol
PVDF	Polyvinylidene fluoride
P(VDF-TrFE)	Poly(vinylidene fluoride-trifluoroethylene
rGO	Reduced graphene oxide
ROS	Reactive oxygen species
SAN	Sinoatrial node
TENG	Triboelectric nanogenerator
VSMC	Vascular smooth muscle cell
